# Mechanistic insights into TTLL11 polyglutamylase–mediated primary tubulin chain elongation

**DOI:** 10.1126/sciadv.adw1561

**Published:** 2025-08-20

**Authors:** Jana Campbell, Miroslava Vosahlikova, Samar Ismail, Margareta Volnikova, Lucia Motlova, Julia Kudlacova, Kseniya Ustinova, Ivan Snajdr, Zora Novakova, Miroslav Basta, Irina Gutsche, Marie-Jo Moutin, Ambroise Desfosses, Cyril Barinka

**Affiliations:** ^1^ Institute of Biotechnology of the Czech Academy of Sciences, BIOCEV, Prumyslova 595, Vestec, Czech Republic.; ^2^ Department of Biochemistry, Faculty of Science, Charles University, Albertov 6, Prague, Czech Republic.; ^3^ Grenoble Institute Neurosciences, University Grenoble Alpes, Inserm U1216, CNRS, 38000 Grenoble, France.; ^4^ Institute of Organic Chemistry and Biochemistry of the Czech Academy of Sciences, Flemingovo n. 2, Prague, Czech Republic.; ^5^ Institut de Biologie Structurale, Université Grenoble Alpes, CEA, CNRS (IBS), 38044 Grenoble, France.; ^6^ Department of Chemistry, Umeå University, SE-901 87 Umeå, Sweden.

## Abstract

Microtubules (MTs) undergo diverse posttranslational modifications that regulate their structural and functional properties. Among these, polyglutamylation—a dominant and conserved modification targeting unstructured tubulin C-terminal tails—plays a pivotal role in defining the tubulin code. Here, we describe a mechanism by which tubulin tyrosine ligase–like 11 (TTLL11) expands and diversifies the code. Cryo–electron microscopy revealed a unique bipartite MT recognition strategy wherein TTLL11 binding and catalytic domains engage adjacent MT protofilaments. Biochemical and cellular assays identified previously uncharacterized polyglutamylation patterns, showing that TTLL11 directly extends the primary polypeptide chains of α- and β-tubulin in vitro, challenging the prevailing paradigms emphasizing lateral branching. Moreover, cell-based and in vivo data suggest a cross-talk between polyglutamylation and the detyrosination/tyrosination cycle likely linked to the TTLL11-mediated elongation of the primary α-tubulin chain. These findings unveil an unrecognized layer of complexity within the tubulin code and offer mechanistic insights into the molecular basis of functional specialization of MT cytoskeleton.

## Teaser

TTLL11 polyglutamylase modifies tubulin by directly elongating the primary polypeptide chains of both α- and β-tubulin.

## INTRODUCTION

The cytoskeleton functions as both a structural scaffold and a dynamic system that drives key cellular processes, including cell division and intracellular transport. Microtubules (MTs), composed of α- and β-tubulin, are central to this framework. Although tubulin subunits are structurally highly conserved across species (especially within their structured cores), diversity arises from multiple isoforms encoded by distinct genes with tissue-specific expression patterns, with most variability observed in sequences of C-terminal tails (*1*). This complexity is further expanded by a range of posttranslational modifications (PTMs), which influence MT stability, interactions, and function. Collectively, these isoform variations and PTMs constitute the tubulin code (*2*).

**Fig. 1.**
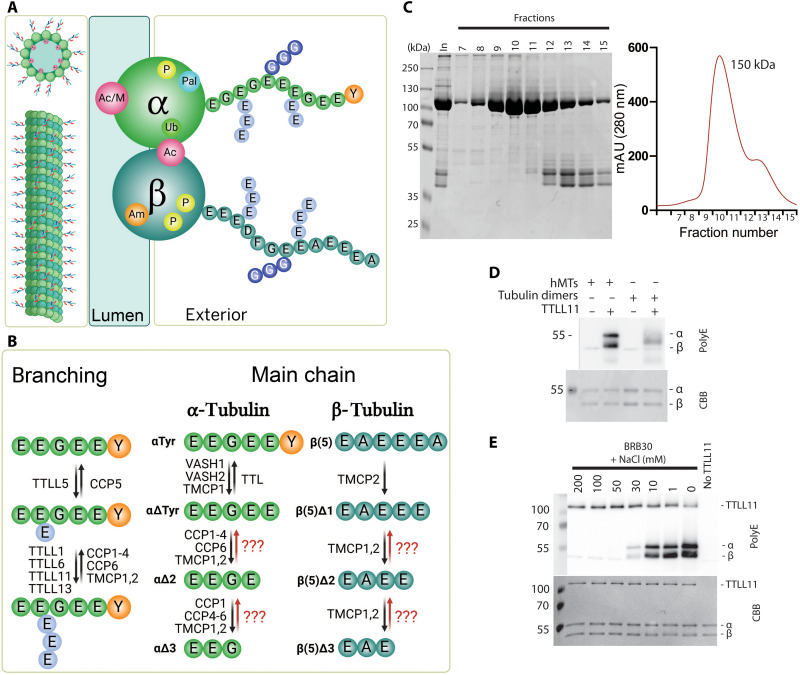
The tubulin code, TTLL11 purification, and tubulin polyglutamylation. (**A**) Posttranslational modifications defining the tubulin code are found at structured tubulin cores [acetylation (Ac), methylation (M), phosphorylation (P), amination (Am), palmitoylation (Pal), and ubiquitination (Ub)] and at disordered C-terminal tails [glycylation (G) and glutamylation (E)]. Created in BioRender. Barinka, C. (2025) https://BioRender.com/r4tzisw. (**B**) Glutamylases of the TTLL family catalyze branching and elongation of polyglutamate chains, which are removed by CCPs and TMCPs. The reversible detyrosination/tyrosination cycle is mediated by TTL, VASHs, and TMCP1. CCPs and/or TMCPs truncate the C termini of tubulin primary chains, yielding irreversible “dead-end” tubulin variants. Created in BioRender. Barinka, C. (2025) https://BioRender.com/baley9y. (**C**) TTLL11 purification. Size-exclusion chromatogram and corresponding Coomassie brilliant blue (CBB)–stained polyacrylamide gel electrophoresis (PAGE) gel from purification of human TTLL11 (hTTLL11) expressed in human embryonic kidney (HEK) 293T cells. The main peak corresponds to a monomeric TTLL11 species (~150 kDa). mAU, milli-absorbance unit. (**D**) TTLL11 prefers MTs over tubulin dimers, polyglutamylating both α- and β-tubulins. Western blot (WB) visualized with the polyE antibody (top) together with the CBB-stained PAGE gel as protein loading control (bottom). hMTs, human microtubules. (**E**) TTLL11 glutamylation activity correlates inversely with ionic strength. TTLL11 efficiently polyglutamylates MTs in low ionic strength buffers (BRB30 + 1, 10, 30, 50, 100, and 200 mM NaCl) with reduced activity observed in increasing salt concentrations. The WB upper ~120-kDa band represents autoglutamylated TTLL11. The CBB-stained gel as a loading control.

Several PTMs, including acetylation, methylation, and phosphorylation, are confined to the tubulin structured core. However, when considering the external surface of MTs and its interactions with molecular effectors, modifications to the unstructured C-terminal tails of tubulin may exert a more pronounced influence, given their prominent exposure, size, and substantial negative charge (Fig. 1A). A genetically encoded C-terminal tyrosine of the α-tubulin primary polypeptide chain is removed by either the vasohibin/small vasohibin–binding peptide complex (VASH/SVBP) (*3*, *4*) or tubulin metallocarboxypeptidase 1 (TMCP1), creating the detyrosinated α-tubulin (αΔTyr) variant (*5*, *6*). The two remaining terminal glutamates can be further trimmed by the action of cytosolic carboxypeptidases (CCPs) (*7*, *8*) or TMCPs (*6*), generating αΔ2 and αΔ3 tubulins. While the removal of the C-terminal tyrosine can be reversed by tubulin tyrosine ligase (TTL) (*9*), the deletion of the terminal glutamates is considered irreversible (*10*). Truncations of the primary polypeptide chain by TMCP2 have also been reported for several β-tubulin isoforms (*6*, *11*). As in the case of αΔ2/αΔ3 tubulins, no rescue of the truncated β chains has been reported in the literature (Fig. 1B).

In addition to primary chain truncations, α- and β-tubulin tails can be laterally branched by polyglutamate chains attached to the γ-carboxyl group of internal glutamate residues (fig. S1). Glutamylation is evolutionarily conserved from ciliates to humans; it is a hallmark of differentiated cells, such as neurons, and is predominantly found in tubulin-rich substructures including axons, dendrites, cilia, flagella, and mitotic spindle. Expectedly, glutamylation homeostasis is tightly regulated, as it is critical for normal cell physiology and aberrant polyglutamylation is linked to neurodegeneration (*7*), ciliopathies (*12*, *13*), immune response defects (*14*), and cancers (*15*–*17*).

Two principal glutamylation steps, branching and elongation, are catalyzed by several members of the TTL–like (TTLL) family, each with distinct substrate specificities (Fig. 1B) (*18*). While tubulin is the best-studied TTLL substrate, polyglutamylation has been reported to modulate the physiological functions of other protein targets such as Disheveled, cyclic guanosine 3′,5′-monophosphate (GMP)–adenosine 5′-monophosphate synthase, and histone chaperones (*14*, *19*, *20*). Moreover, existing data suggest that glutamylation might be more widespread than currently acknowledged, and the TTLL11 enzyme can play a prominent role in polyglutamylation of nontubulin substrates (*21*). Depending on the organism and the cell type, TTLL11 is localized in the nucleus in human fibroblasts and HeLa cells (*22*), but it was observed in the cilium in *Caenorhabditis elegans* (*23*) and the basal body of Madin-Darby canine kidney cells (*18*). TTLL11 is critical for glutamylation of the mitotic spindle, and its silencing decreases the chromosome segregation fidelity associated with cancer (*17*). At the organism level, TTLL11 mutations affect skeletal development in humans, and these observations were replicated in zebra fish, where TTLL11 mutations led to mostly fatal spine curvature defects (*24*).

By combining cryo–electron microscopy (cryo-EM) and functional assays, this study uncovers an unanticipated type of tubulin glutamylation pattern, where TTLL11 extends the primary polypeptide chains of both α- and β-tubulin with specificity driven by the amino acid composition of the respective C-terminal residue. It further reveals a cross-talk between other tubulin-modifying enzymes and TTLL11 and specifically between the vital detyrosination/tyrosination cycle of α-tubulin and TTLL11 polyglutamylase. This discovery challenges the existing understanding of tubulin modifications, which primarily emphasizes lateral branching polyglutamylation. Our work thus substantially advances the field by indicating an expansion of the known repertoire of modifications within the tubulin code.

## RESULTS

### TTLL11 polyglutamylates MTs at both α- and β-tubulin chains

To elucidate the substrate specificity of TTLL11, we expressed and purified the human enzyme [human TTLL11 (hTTLL11); Fig. 1C] and assayed its competency to modify tubulins isolated from human embryonic kidney (HEK) 293T cells. Our results demonstrate that TTLL11 preferentially polyglutamylates polymerized MTs rather than free tubulin (Fig. 1D). Consequently, all subsequent in vitro assays were conducted using MTs. Notably, TTLL11 activity exhibited sensitivity to the ionic strength of the buffer, with polyglutamylation levels increasing with decreasing salt concentration (Fig. 1E). Our findings also reveal that both α and β subunits are polyglutamylated by hTTLL11 in vitro, indicating that the enzyme exhibits a broad substrate specificity that was not previously recognized (*18*, *21*). The lack of hTTLL11 selectivity for either α- or β-tubulin thus contrasts with the substrate specificity of TTLL6 and TTLL7 paralogs with ascribed preferences for α and β chains, respectively (*25*, *26*).

### TTLL11 simultaneously engages adjacent MT protofilaments

**Fig. 2.**
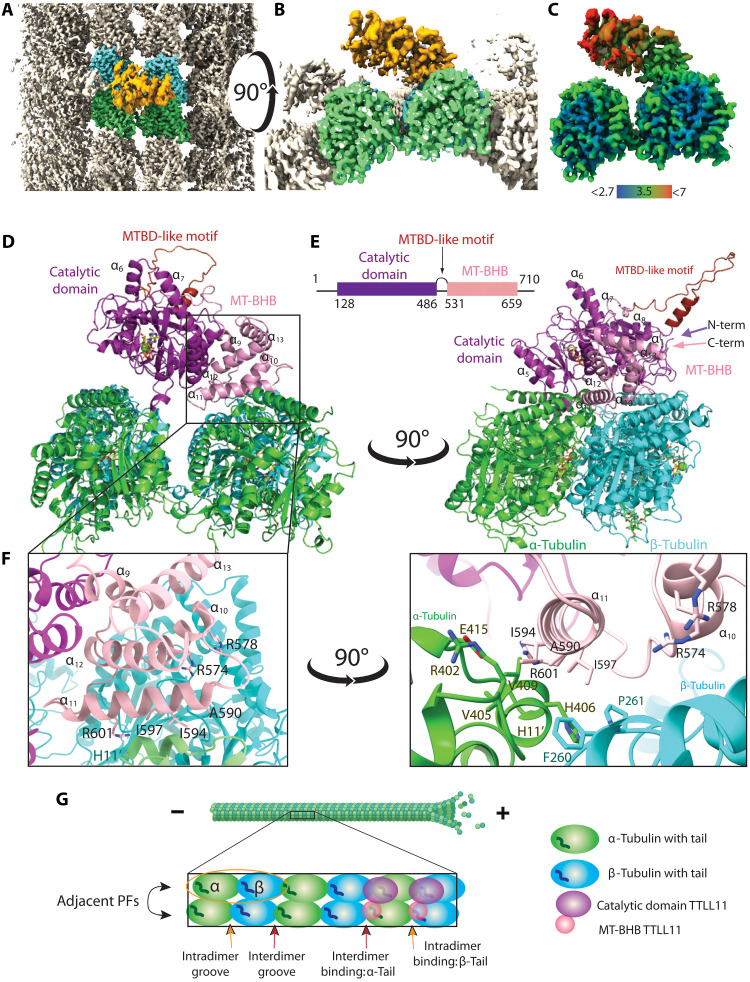
The cryo-EM reconstruction of the TTLL11/MT complex. (**A** and **B**) The cryo-EM map shows TTLL11 binding to the MT lattice. TTLL11 in gold, MT in gray, and the central α- and β-tubulin dimers in green and cyan, respectively [(A): top view and (B): side view]. (**C**) Cryo-EM map colored by local resolution, ranging from 2.7 Å (blue; tubulin core) to more than 7 Å (red; distal TTLL11 catalytic domain). (**D**) A cartoon model of the TTLL11/MT complex. TTLL11 binds the α/β intradimer interface via its MT-BHB (pink; 9HQ4), positioning the catalytic domain (magenta) over the β-tubulin C-tail of an adjacent protofilament (PF). The helix-loop-helix motif [hot pink; MT-binding domain (MTBD)–like] is structurally analogous to TTLL6/7 binding domains but is not implicated in MT interactions. (**E**) Schematic representation of the TTLL11 structure. The catalytic domain (purple) and the MT-BHB (pink) are connected via the unstructured MTBD-like motif. Intrinsically disordered N and C termini are shown as black lines. (**F**) Details of the TTLL11/MT interface. Zoomed view of interface between MT-BHB and tubulin dimer [helix H11′ and loop between S8 and H10′ of α- and β-tubulin, respectively (*29*)]. (F). Residues A590, I594, I597, and R601 (helix α_11_) and R574/R578 (helix α_10_) of TTLL11 contribute prominently to the mixed ionic/hydrophobic interface with MTs. (**G**) Schematic representation of MT PFs and allowed TTLL11 binding poses. MTs are polar tubular assemblies of α/β-tubulin dimers, with β-tubulin on the (plus)-end. Dimers (yellow oval) form PFs, stacked side by side in a circular arrangement. Intradimer grooves (yellow arrow) are located between α- and β-tubulin in dimers, while interdimer grooves (red arrow) separate adjacent dimers. TTLL11 MT-BHB (pink) can bind both the inter- or intradimer grooves, positioning thus the catalytic domain (purple) over the α- or β-tubulin tail, respectively. Created in BioRender. Barinka, C. (2025) https://BioRender.com/sr4q7do.

To provide the structural basis for TTLL11 substrate recognition and interpret potential preferences for polymerized MTs and α- versus β-tubulin chains, we determined the cryo-EM structure of hTTLL11 (residues 128 to 657) in the complex with double-stabilized MTs (dsMTs) isolated from HEK293T cells. The final cryo-EM map has a nominal resolution of 3.28 Å with local resolution estimate up to 2.8 Å (Fig. 2, A to C, and fig. S2, A to C). The well-resolved map facilitated the unambiguous assignment of individual tubulin protomers (fig. S2D) and the construction of a high-confidence atomic model comprising the unconventional MT-binding helix bundle (MT-BHB) of hTTLL11 (residues C531 to R659; Fig. 2, D and E, and fig. S2, E and F). The MT-BHB, which does not have any homology counterparts in MT-binding motifs of other members of the TTLL family, represents a critical component of the TTLL11/MT interaction interface (Fig. 2F and figs. S2G and S3, A to C) (*27*, *28*). Although the density representing the TTLL11 catalytic core (residues P128 to P486) is less resolved, it was still possible to reliably dock the AlphaFold TTLL11 model into the cryo-EM map. The following segment (K488 to V535) connecting the catalytic domain to the MT-BHB is not visible in the map and therefore not included in the model, nor are the intrinsically disordered N and C termini, which are also missing from the cryo-EM map. Together, the cryo-EM data indicate that while the MT-BHB is rigidly docked onto the MT surface, the TTLL11 catalytic core (together with intrinsically disordered regions) and tubulin C-terminal tails are inherently flexible and can adopt a wide range of conformations.

Two unexpected findings emerged from our cryo-EM reconstruction. First, TTLL11 recognizes MTs in a unique bipartite pattern, where the MT-BHB and the catalytic domain engage adjacent protofilaments (PFs). This stands in stark contrast with the structures of TTLL6 and TTLL7 complexes, where both MT binding and polyglutamylation are confined to the same PF (fig. S4) (*25*, *28*). Second, the MT-BHB interacts with residues of the longitudinal intradimer α/β groove, positioning thus the catalytic TTLL11 domain atop the C-terminal tail of the β subunit of the adjacent PF (Fig. 2, F and G, and fig. S5, A to C). These findings are consistent with the biochemical data showing that the tubulin tetramer within the MT lattice, but not the tubulin dimer, represents the minimum TTLL11 recognition motif (Fig. 1D). At the same time, the positioning of the catalytic domain above the β subunit C-terminal tail would point toward a preference for polyglutamylation of β-tubulin. However, both α- and β-tubulin chains of the tubulin isolated from HEK293T cells are polyglutamylated to a similar extent in vitro (Fig. 1, D and E).

### The MT-BHB does not discriminate between intra- and interdimer tubulin interfaces

Sequence alignment and structural models indicate that the MT-BHB of TTLL11 is distinct from known or presumed MT-binding segments of other TTLL family members (fig. S4). The structured part of the MT-BHB forms a five-helix bundle (comprising helices α_9_ through α_13_) that is in apposition to the catalytic domain with a shared interface of ~1500 Å^2^. The cryo-EM structure revealed that the primary MT interaction motif of MT-BHB involves two amphipathic helices encompassing amino acids T571 to C580 (helix α_10_) and M589 to R601 (helix α_11_; Fig. 2, E and F). The positively charged R574 and R578 (α_10_) and R601 (α_11_) are oriented toward the negatively charged tubulin surface to mediate putative ionic interactions (fig. S3A). However, the key interaction motif involves the hydrophobic face of the α_11_ helix comprising A590, I594, and I597 that is engaged by residues of the H11′ helix of α-tubulin and the side chains of F260/P261 of the β subunit (Fig. 2F) (*29*). The pivotal contribution of the side chain of I594, which is inserted into the complementary tubulin hydrophobic pocket delineated by the side chains of R402, V405, H406, V409, and E415 (Fig. 2F and figs. S3, A and B, and S5, D and E), was corroborated by side-directed mutagenesis and in vitro experiments (see below).

Given the promiscuity of TTLL11 toward α- and β-tubulin tails, we further analyzed the sequence and structural conservation of tubulin residues implicated in interactions with MT-BHB. Overall, the sequence and structural conservation of the core of individual tubulin isoforms and between α- and β-tubulins (fig. S5, D and E) indicates that while MT-BHB is conceivably the primary MT recognition motif anchoring TTLL11 to the MT surface, it alone is unable to discriminate between intra- and interdimer groove interfaces of tubulin within the MT lattice. Consequently, isolated MT-BHB cannot dictate TTLL11 preferences for either α- or β-protomer, and TTLL11 selectivity (or lack thereof) for either tubulin protomer requires additional inputs from the catalytic domain.

### Bipartite engagement is essential for efficient MT binding and polyglutamylation by TTLL11

**Fig. 3.**
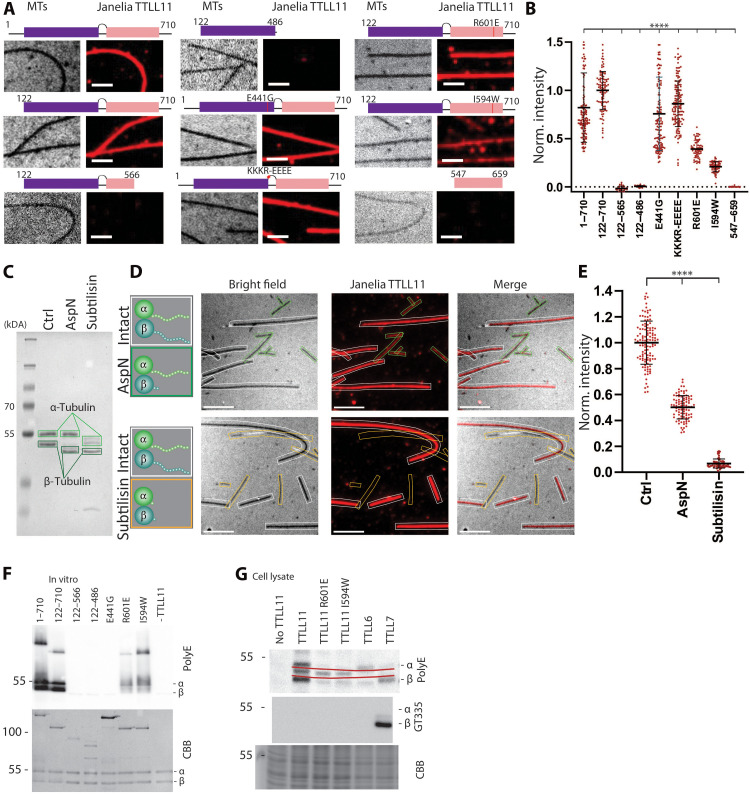
Cross-talk between the catalytic domain and MT-BHB is critical for MT binding and polyglutamylation by TTLL11. (**A**) TTLL11 binding to pMTs analyzed using TIRF microscopy. The TTLL11 representation shows the catalytic domain (purple), MT-BHB (pink), and site-specific mutations (described in fig. S6). MTs were attached to coverslips and incubated with 100 nM Janelia Fluor 549–labeled TTLL11 variants (red). Scale bars, 2 μm. (**B**) Quantification of TIRF images for full-length (FL) TTLL11, the N-terminally truncated variant (122 to 710), the catalytically inactive E441G, and the KKKR-EEEE mutant show binding with similar intensity. Deleting the MT-BHB or catalytic domains abolishes TTLL11 binding. Mutations in TTLL11 helix α_11_ (R601E and I594W) reduced binding by 60 and 80%, respectively. Norm., normalized. (**C** to **E**) Tubulin C-tails are critical for TTLL11 interactions. (C) CBB-stained gel of truncated MTs. AspN truncates the β-tubulin, and subtilisin truncates both α- and β-tubulin C-tails. TIRF microscopy images (D) and quantification (E) show that AspN-treated MTs exhibit ~50% reduction in TTLL11 binding, while the complete loss of binding is observed for subtilisin-treated MTs. Data are shown as mean fluorescent intensity from *n* = 2 replicates with 114, 93, and 58 MTs quantified in each sample. Statistical significance: Unpaired *t* test with Welch correction; ****P < 0.0001. The black bar: Median value with 95% confidence interval. Scale bars, 5 μm. Ctrl, control. Created in BioRender. Barinka, C. (2025) https://BioRender.com/sr4q7do. (**F**) In vitro polyglutamylation of MTs from HEK293T cells by TTLL11 variants, analyzed by WB, confirms activity for FL and 122 to 710. Variants lacking the MT-BHB domain show no activity. I594W and R601E mutants show reduced activity. (**G**) Assay in HEK293T cells transfected with TTLL11 variants shows polyglutamylation consistent with in vitro findings. TTLL6 and TTLL7 show specificity for α- and β-tubulin, respectively. An unidentified TTLL11 substrate, represented by a polyE-stained band in between α- and β-tubulin (between red lines), is glutamylated regardless of the MT-BHB mutations. Only TTLL7 shows a signal of the GT335 antibody specific for branching.

To evaluate if and how the interplay between the catalytic domain and MT-BHB influences TTLL11 interactions with MTs, we generated a series of TTLL11 variants (fig. S6, A and B). These variants were assayed for their ability to bind MTs using total internal reflection fluorescence (TIRF) microscopy and modify MTs using the polyglutamylation assay. For the TIRF binding assay, porcine dsMTs were immobilized onto coverslips, and the binding of fluorescently labeled TTLL11 variants was quantified. While the strong fluorescence signal was observed for the full-length TTLL11 and variants lacking the N-terminal region (residues M1 to G121), neither the isolated MT-BHB (residues R547 to R659) nor the isolated catalytic domain (residues E122 to P486) was able to bind MTs (Fig. 3, A and B). Furthermore, the importance of MT-BHB (and its cross-talk with the catalytic domain) was underscored by the analysis of the I594W and R601E mutants, which were designed to impair interactions with MTs based on our cryo-EM structure. Here, the fluorescence signal intensity was reduced by 60 and 80% for the R601E and I594W variants, respectively. Only a small decrease was observed for the KKKR-EEEE mutant (K_488_KKR_491_ to E_488_EEE_491_), where the mutation is located within the MT-binding domain (MTBD)–like motif. Similarly to the glutamylase activity, the TTLL11 binding to the MT surface was also dependent on the ionic strength of the assay buffer (fig. S6, C and D).

In a complementary set of experiments, we generated modified MTs in which the C-tails of dsMTs were removed by treatment with AspN (β-tail only) or subtilisin (both α- and β-tails; Fig. 3C). At 100 nM concentration, the fluorescence TTLL11 signal for AspN-treated MTs was ~50% lower compared to intact MTs, and no binding was observed for subtilisin-treated MTs. The C-terminal tail of both tubulin isotypes is essential for anchoring TTLL11 to MTs, and the monodentate binding mediated by MT-BHB is not sufficient. The combined contribution of both TTLL11 domains is thus essential for effective MT binding (Fig. 3, D and E).

The TIRF experiments were further corroborated in vitro and by cell-based assays where we evaluated MT polyglutamylation by TTLL11 variants (Fig. 3, F and G). While wild-type (WT) TTLL11 showed pronounced tubulin polyglutamylation, the presence of the inactive E441G mutant or the isolated catalytic domain did not change polyglutamylation beyond background levels. Similarly, tubulin polyglutamylation was markedly impaired in the case of the I594W and R601E mutants. Combined, these results show that the lower binding affinity of TTLL11 variants is directly translated into less efficient polyglutamylation of the target tubulin substrate.

### TTLL11 expands the tubulin code by extending the primary tubulin chains in vitro

**Fig. 4.**
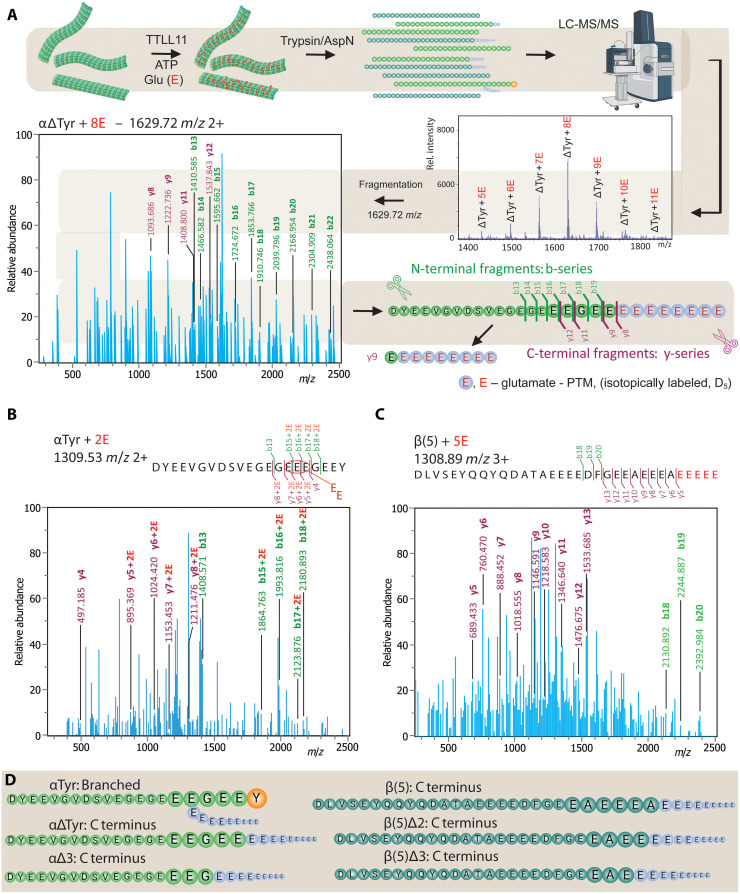
Identification of polyglutamylation sites within tubulin sequences using the LC-MS/MS pipeline. (**A**) Schematic representation of the LC-MS/MS pipeline. MTs are incubated with TTLL11 in vitro in a reaction mixture supplemented with perdeuterated D_5_-glutamate. Following digestion with either trypsin (α-tubulin C-tails) or AspN (β-tubulin C-tails), resulting peptides are analyzed by LC-MS/MS for the presence of (poly)glutamylated species. For each of the (poly)glutamylated peptides, MS/MS fragmentation is performed to identify the site(s) of attachment of the polyglutamate chain(s), based on the presence of the b- and y-series of peptides, which correspond to N- and C-terminal fragments, respectively. The black lines mark specific mass/charge ratio (*m/z*) peaks of notable fragments used for the peptide identification. (**B** and **C**) Illustrative examples of MS/MS spectra of polyglutamylated tubulin species. The LC-MS/MS pipeline was used to analyze tubulin variants that were polyglutamylated by TTLL11 in vitro. Peaks of interest observed in b- and y-series are highlighted in green and magenta, respectively. This approach enabled the unequivocal assignment of attachment sites for polyglutamate chains within the tubulin sequence. Rel., relative. (**D**) Summary of tubulin sites preferentially polyglutamylated by TTLL11. The combined LC-MS/MS data reveal that the tyrosinated α-tubulin (αTyr) variant is branched (and extended) at positions E445, E446, and E447, albeit at marginal levels. In contrast, the remaining tubulin variants are preferentially polyglutamylated at their C termini by the direct extension of the primary polypeptide chain. The corresponding MS and MS/MS spectra and quantifications are shown as figs. S9 to S12. Created in BioRender. Barinka, C. (2025) https://BioRender.com/sr4q7do.

While Western blotting is a valuable tool for qualitative analysis of polyglutamylation in cells and in vitro, it does not allow for the identification of polyglutamylation attachment sites and polyglutamate chain length and connectivity. To overcome these limitations, we implemented a liquid chromatography–tandem mass spectrometry (LC-MS/MS) pipeline that enabled us to gain detailed qualitative and quantitative insights into tubulin polyglutamylation by TTLL11. For MS/MS experiments, samples were digested with the trypsin/LysC mix or AspN for the analysis of the C terminus of α- and β-tubulin, respectively. MS data were used for quantification, while MS/MS spectra provided information about the positions of lateral branching points and linkage chemistry. Accurate identification of fragment peaks was facilitated by exploiting isotopically labeled perdeuterated D_5_- and ^18^O-glutamates, which are crucial for determining the exact position and bonding pattern of glutamate chains (Fig. 4A).

In our original set of experiments, we used mostly unmodified native tubulin isolated from HEK293T cells, where TUBA1A and TUBA1B isoforms, sharing identical C-terminal sequences, account for more than 80% of all α-tubulins (figs. S7, A and B, and S8, A and B) (*30*). Furthermore, tyrosinated α-tubulin (αTyr) represents ~80%, while αΔTyr and αΔ2 variants are much less populated (~15%). The remaining 5% minority species included C-tails with one or two glutamate residues added at unknown position(s) within the sequence. For β-tubulins, TUBB5 and TUBB4B were the most populated isoforms (~80%), followed by lower amounts of TUBB2A/B (figs. S7, A and B, and S8C) (*30*). Less than 5% of native β-tubulins were modified by an extra glutamate residue.

Upon incubation with purified TTLL11, we observed massive polyglutamylation of predominantly αΔTyr and αΔ2 variants with up to 11 glutamate residues added. Notably, the MS/MS fragmentation spectra revealed that the glutamate residues are primarily attached to the very C terminus of the main polypeptide, thus comprising a previously unrecognized type of tubulin modification (Fig. 4A and fig. S9A). The αTyr variant was not C-terminally extended but rather laterally branched at E445, E446, or E447, albeit at marginal levels (Fig. 4B).

For β-tubulin, after incubation with TTLL11 in vitro, up to 27 glutamate residues were attached to the tails, and the MS/MS spectra revealed the direct extension of the native C termini, where polyglutamate chains were attached to the terminal alanine residue shared by the TUBB5/TUBB4B isoforms (Fig. 4, C and D, and fig. S9, B and C).

### TTLL11 and TTLL6 elongate the primary tubulin chain even in the presence of lateral branching

To analyze TTLL11 preferences for elongation of existing laterally branched chains versus extending the tubulin main chain, we used tubulins isolated from porcine brain as substrates for in vitro reconstitution assays. In contrast to unmodified HEK293T tubulin, porcine tubulin contains a diverse spectrum of modified tubulins with mono- and polyglutamylated species representing ~60% of the total α-tubulins. In addition, the αΔTyr and αΔ2 variants are also more abundant, accounting for ~60 and 20% of α chains, respectively (fig. S10A). After incubation with purified TTLL11, MS/MS analysis revealed the presence of tubulin species directly elongated at the C terminus of αΔTyr and elongation at preexisting branching points (fig. S10, B to E). Unfortunately, the complexity of the substrate and product species in the reaction mixture did not allow for their precise quantification, as they can only be distinguished on the fragmentation spectrum level due to their isobaric molecular weights (fig. S10, D and E).

The above findings raised an interesting idea that other elongases may also be capable of extending the primary tubulin chain, either independently or in conjunction with the elongation of the preexisting glutamate lateral branches (*26*). To test this hypothesis, we used MS/MS to analyze tubulin polyglutamylation by TTLL6 in vitro, which preference for αΔTyr is known (*26*). Our data demonstrate that TTLL6 is capable of elongating both the branching points and the main chain of the αΔTyr variant (fig. S11, A to C). In accordance with the known TTLL6 preferences, marginal elongation of the C termini of β-tubulins was observed (fig. S11D). Unexpectedly, direct extension of the tubulin main chain may thus represent a common enzymatic activity of TTLL elongases, a possibility raised by assessing TTLL6 substrate preferences at the peptide level (*26*) but never reported and studied in the context of MTs, as the major physiological target of TTLLs.

**Fig. 5.**
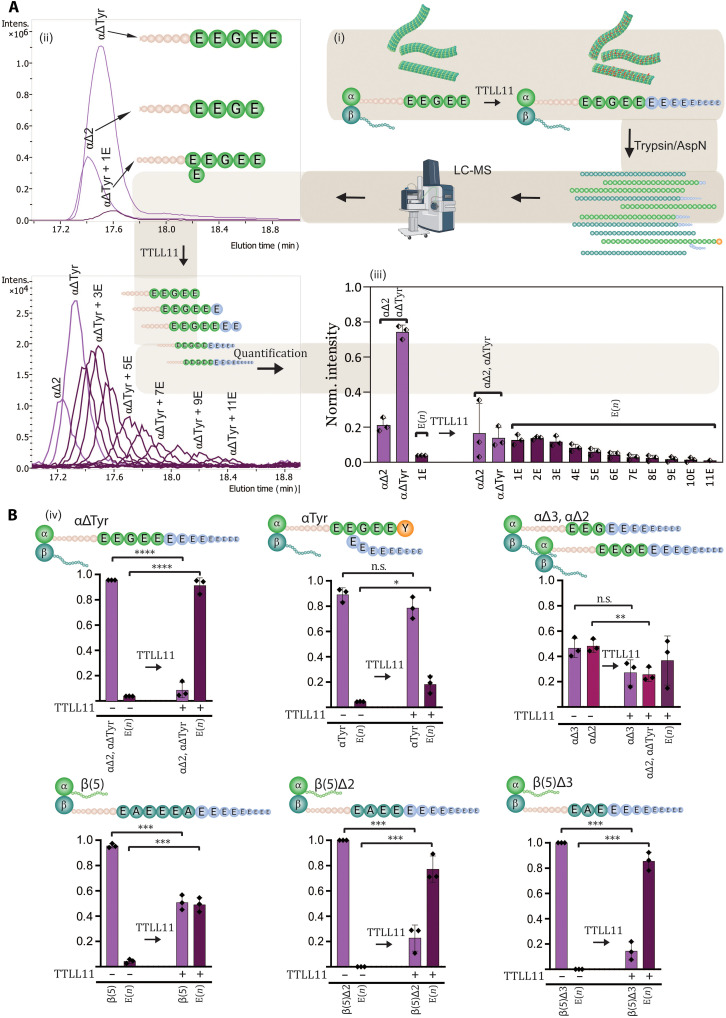
Quantitative analysis of tubulin polyglutamylation by TTLL11. (**A**) An illustrative example of the quantification of tubulin C-tail polyglutamylation by LC-MS. (i) Sequences of the C-terminal tryptic peptide of αΔTyr before and after TTLL11-mediated polyglutamylation. (ii) The extracted ion chromatograms show peaks corresponding to the peptide without glutamylation and with 0 to 11 glutamate residues attached. Intens., intensity. (iii) Quantification of the individual C-tail peptides with different polyglutamate chains. (iv) A summary graph showing the normalized MS intensities before and after TTLL11 treatment derived from (ii) and (iii). (**B**) Summary quantification of tubulin polyglutamylation by TTLL11. Tubulin variants (in the form of MTs) were incubated with TTLL11, and intensities of peptides before and after reaction were summed. The data are shown as the normalized MS intensities, where the sum of intensities of the peptide pool before and after TTLL11 treatment equals 1 (*n* = 3; statistical significance was determined using an unpaired *t* test; *P < 0.05, **P < 0.01, ***P < 0.001, and ****P < 0.0001). While only marginal branched polyglutamylation is observed for the αTyr variant, the remaining variants are efficiently polyglutamylated with conversions more than 50% of the original tubulin (>80 and > 90% for β(5)Δ2/β(5)Δ3 and αΔTyr, respectively). Detailed quantification of individual glutamylated peptides is shown in fig. S12. n.s., not significant. Created in BioRender. Barinka, C. (2025) https://BioRender.com/sr4q7do.

Together, these data demonstrate that TTLL11 elongates both α- and β-tubulin main chains in vitro, thereby likely expanding a portfolio of modifications that comprise the tubulin code (Fig. 5B). While TTLL11 can, in principle, also laterally branch the internal C-tail glutamates of α-tubulins, this reaction is extremely inefficient, and we believe that it might be an artifact of in vitro reaction conditions. When analyzing the tubulin polyglutamylation patterns in reactions using ^18^O-labeled glutamate, the MS spectra were dominated by peptide envelopes retaining the ^18^O isotope, thus providing further experimental evidence that the peptide bonds are formed via the α-carboxyl group linkage rather than γ-carboxylate lateral branching (fig. S11, E and F). Therefore, we propose that the enzyme acts exclusively as an elongase under physiological conditions (*26*), corroborating its proposed functions in an earlier study done in HeLa cells (*18*).

### Sequences of C-terminal tails dictate TTLL11 preferences for α- versus β-tubulins

Our data reveal that TTLL11 can extend the C terminus of both α- and β-tubulins and that the C-terminal glutamate is not strictly required for the substrate to be modified. At the same time, when comparing the efficiency of polyglutamylation of αTyr versus αΔTyr (Fig. 5, A and B, and fig. S12, A and B), it is evident that TTLL11 discriminates between amino acid sequences of target substrates. To quantitatively analyze TTLL11 preferences for naturally occurring tubulin variants with different C termini, we treated HEK293T tubulin with recombinant tubulin-modifying enzymes, yielding αTyr-, αΔTyr-, αΔ2-, αΔ3-, βΔ2-, and βΔ3-enriched tubulin fractions for in vitro reconstitution experiments (figs. S7A and S8, A to C).

For α-tubulin variants, the main chain of either αΔTyr, αΔ2, or αΔ3 variants was directly extended with the reaction efficiency decreasing in the order of αΔTyr, αΔ2 > αΔ3, with 90 and 20% of the substrate modified, respectively (Fig. 5, A and B, and fig. S12, A to C). These results are particularly exciting with respect to the αΔ2 and αΔ3 variants, which were believed to be the dead-end products of α-tubulin C-tail modifications that could not be reverted to the native genetically encoded sequences (*10*, *11*). Accordingly, TTLL11 can rescue αΔ2 and αΔ3 variants, forming αΔTyr that could either be tyrosinated by TTL or generate an unconventional C-terminal polyglutamate chain (Fig. 4D and fig. S9A).

**Fig. 6.**
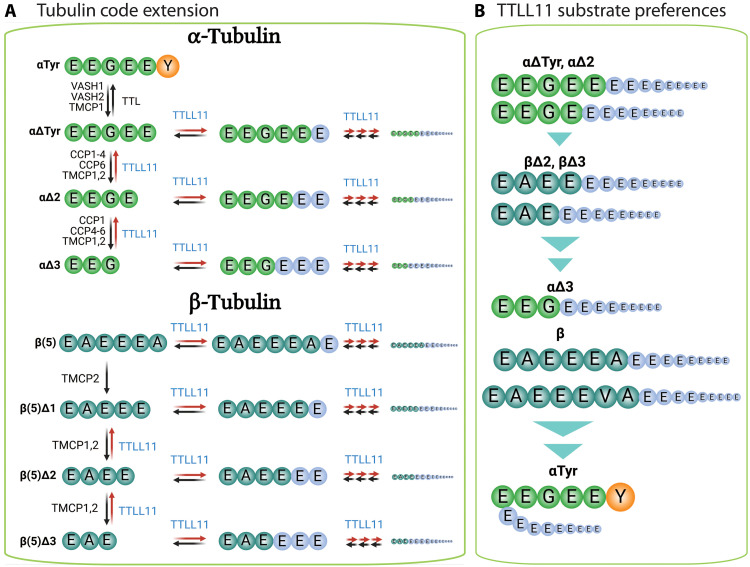
Expansion of the tubulin code by TTLL11-mediated polyglutamylation. (**A**) TTLL11 activity expands the tubulin code. Polyglutamylation at the C termini of the tubulin primary chains can rescue and recycle truncated tubulin variants that were previously considered irreversible end-products. In addition, the main chain extension creates uncharacterized tubulin variants that could potentially be associated with hitherto unidentified physiological functions. Created in BioRender. Barinka, C. (2025) https://BioRender.com/v63178a. (**B**) An overview of TTLL11 substrate specificity. TTLL11 preferentially elongates the main chain of α- and β-tubulins with selectivity defined by the characteristics of the terminal amino acid. The ultimate glutamate residues are favored, indicating that αΔTyr, αΔ2, βΔ2, and βΔ3 are optimal physiological substrates. As small aliphatic and hydrophobic residues are also permitted, αΔ3 and intact β-tubulins are also polyglutamylated with acceptable efficiency. The presence of the C-terminal Tyr/Phe prevents direct extension of the α-tubulin primary chain. Created in BioRender. Barinka, C. (2025) https://BioRender.com/d4thvtt.

Recently identified TMCP enzymes were reported to remove residues from the C terminus of β-tubulin, generating thus βΔ2 and βΔ3 variants (*6*). While native β-tubulins harboring the C-terminal alanine were glutamylated to ~50%, the polyglutamylation efficiency of βΔ2 and βΔ3 variants was almost 80% and thus comparable to results observed for αΔTyr (Fig. 5B and fig. S12, D to G). The C-terminal amino acid thus serves as the key determinant of TTLL11 glutamylation activity and determines TTLL11 preference for either α- or β-isotype (Fig. 6).

### Cross-talk between tubulin-modifying enzymes of polyglutamylation and detyrosination

**Fig. 7.**
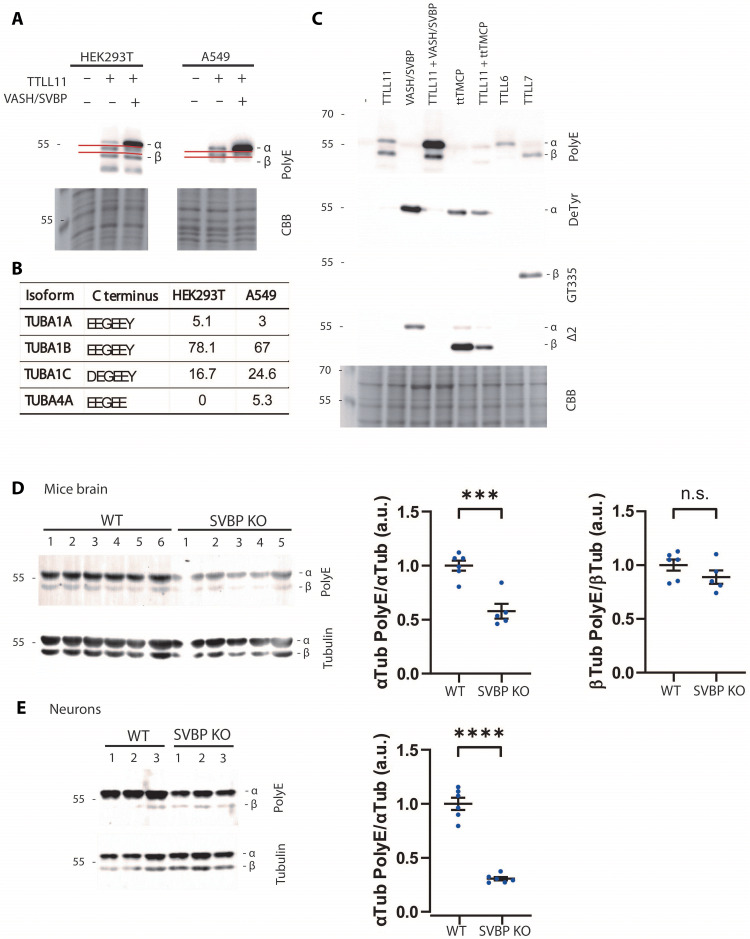
Cross-talk between TTLL11 and tubulin-modifying enzymes. (**A** to **C**) Isoform selectivity of TTLLs in cells. WB (PolyE and deTyrosinated tubulin antibodies) of HEK293T/A549 cell lysates (co)transfected with TTLL11, VASH2/SVBP, ttTMCP, TTLL6, and TTLL7. CBB-stained gel represents loading controls. (A) The ratio of α- versus β-tubulin polyglutamylation partially depends on expression of the TUBA4A isoform lacking the C-terminal tyrosine (expressed in A549 cells) which is a preferred TTLL11 substrate (αΔTyr), leading to higher proportion of glutamylated α-tubulins. Red lines border a band of the unknown TTLL11 substrate. (B) Normalized mRNA transcript abundance of α-tubulin isoforms in HEK293T and A549 cells (*30*). The TUBA4A isoform is only present in A549 cells. (C) VASH/SVBP transfection increases polyglutamylation of α-tubulin. α-Tubulin polyglutamylation in HEK293T cells cotransfected with TTLL11 and VASH/SVBP (creating αΔTyr) is notably increased. ttTMCP reverses TTLL11-mediated polyglutamylation. TTLL6 and TTLL7 polyglutamylate α- and β-tubulin, respectively. GT335 antibody shows that branching is catalyzed only by TTLL7, but not TTLL6 or TTLL11. The overexpression of VASH2/SVBP enables further truncations as visualized by the anti-Δ2 antibody. ttTMCP also creates αΔ2 but preferentially truncates β-tubulin C terminus, creating the β(5)Δ3 variant with the C-terminal sequence (Glu-Ala-Glu) analogous to αΔ2-ending (Glu-Gly-Glu). (**D** and **E**) Tubulin polyglutamylation of WT and SVBP knockout (KO) mice brain and cortical neurons cultured for 8 days in vitro. Representative immunoblots and corresponding quantification of the ratio between (polyE Gre α-tubulin)/(total α-tubulin) and (polyE Gre β-tubulin)/(total β-tubulin) in mice brains (D) and neurons (E). The polyE Gre signal of β-tubulin in neuron lysates was too low for effective quantification. PolyE Gre antibody specificity (fig. S16). Data represent mean ± SEM. *n* = 5 and 6 animals and six independent neuronal differentiation experiments of each genotype. Unpaired *t* test; n.s, ***P < 0.001, and ****P < 0.0001. Comparative analyses of αTyr and αΔTyr are shown in fig. S12. αTub, α-Tubulin; βTub, β-Tubulin.

We first replicated the biochemical in vitro experiments at the cellular level. Upon transfection of HEK293T and A549 cell lines with WT TTLL11, we observed the increase in the polyE signal intensities (Fig. 7, A and B) for both α- and β-tubulins. In line with the low abundance of the αΔTyr variant (HEK293T), the most preferred TTLL11 substrate, the signal for polyglutamylated α-tubulin, was less prominent than β-tubulin. Cell transfections with VASH2/SVBP led to the enrichment of the αΔTyr variant, and a subsequent marked increase of the polyE signal of α-tubulin was observed in cells cotransfected with the combination of TTLL11/VASH2/SVBP (Fig. 7, A to C). We could not observe any enrichment of β-tubulin polyglutamylation upon cell cotransfection with the combination of TTLL11 and *Tetrahymena thermophila* TMCP (ttTMCP; Fig. 7C). It is plausible that while the emergence of the Δβ2 variants would increase TTLL11 polyglutamylation rate toward this substrate as observed in vitro, ttTMCP, which has much higher in vitro hydrolytic activity compared to human enzymes, would simultaneously catalyze the removal of added polyglutamate chain from β-tubulin chains, thus counterbalancing TTLL11 activity. In addition, not unexpectedly, the rate of α/β-tubulin polyglutamylation in a given cell would depend on the spatiotemporal distribution and activity of each of the tubulin-modifying enzymes and their mutual interplay.

Furthermore, we investigated the impact of TTLL11 ectopic expression in hippocampal neurons cultured in vitro. The neurons, which are known to be rich in αΔTyr and αΔ2 variants (*31*) and polyglutamylated tubulins (*32*, *33*), demonstrated a significant loss of αΔ2 together with an increase in polyglutamylation following overexpression of active TTLL11 (fig. S13, A to D). These results offer indirect evidence in support of the concept that TTLL11 polyglutamylates the C terminus of α-tubulin, thereby reversing the αΔ2 to αΔTyr and/or extending the main chain by a C-terminal polyglutamate chain. The signal with GT335 antibody also increased with expression of the active TTLL11 in neurons (fig. S13C), which was not observed in the HEK293T cell line (Fig. 7C). It is unclear at this moment whether the additional branching results directly from TTLL11 activity or whether it is mediated by other TTLLs acting on newly formed polyglutamate chains, such as TTLL7, an efficient branching enzyme (Fig. 7C) with transcripts abundant in hippocampal neurons (*34*). While TTLL11 prefers the elongation reaction, it was also able to initiate a side chain on both α- and β-tubulin in overexpression experiments in HeLa cells (*18*).

Last, we studied whether the loss of VASH/SVBP enzymes in vivo could influence the level of tubulin polyglutamylation in the brain by reducing the availability of αΔTyr and αΔ2, the preferred TTLL11 substrates (fig. S14, A and B). We thus compared the levels of polyglutamylated tubulin in brain extracts and neuronal cultures from WT and SVBP knockout (KO) mice, given that the defective tubulin detyrosination, resulting from the absence of the chaperone SVBP, has a marked impact on the structure and function of the brain in both humans and mice (*35*). In accordance with the result from biochemical and cellular experiments, the data obtained from in vivo studies demonstrated that the absence of VASH/SVBP-detyrosinating enzymes markedly diminishes α-tubulin polyglutamylation in the brain tissue, whereas the polyglutamylation levels of β-tubulin remain unaltered (Fig. 7, D and E).

Our data show that the polyglutamylase activity correlates with that of VASH detyrosinases in both neurons and brain tissue. These findings are consistent with our in vitro results on TTLL11 and, given its abundant expression in these cells and tissues (*34*), may reflect modulation of TTLL11 activity on the α-tubulin primary chain. However, we cannot exclude the contribution of other TTLLs, such as TTLL1, which is also highly expressed and functionally relevant in this context and primarily targets α-tubulin (*36*). Because of its complex multimeric structure (*37*, *38*), TTLL1 has not been thoroughly characterized in vitro, and its capacity to elongate primary tubulin chains remains unknown.

## DISCUSSION

Together with TTLLs 1, 6, 9, and 13, TTLL11 is classified as a glutamate elongase (*18*). The original biochemical findings were later corroborated by comprehensive structural analyses of TTLL6, which identified Q180 as the primary determinant of its selectivity (*26*). The arrangement of the active site of TTLL11 is analogous to TTLL6, including the conservation of Q255 corresponding to Q180 of TTLL6 (fig. S2F). Our MS/MS data confirmed the classification of TTLL11 as an elongase by showing that elongation is much preferred compared to the lateral branching activity and that elongation is likely the only TTLL11 physiologically relevant activity (Fig. 5B). Furthermore, our findings strongly suggest that elongation of tubulin primary chains by TTLL11 is preferred over elongation of preexisting glutamate lateral branches, although the mechanistic basis for this selectivity remains unclear and cannot be derived from the cryo-EM structure.

Earlier TTLL6/7 structures revealed tripartite (or quadripartite) interactions with MTs, involving catalytic domains (brown), MTBDs (orange), and MT-binding helices (dark red) (fig. S4). Moreover, an additional interaction motif must include tubulin C-tails and the residues lining the active sites of TTLL6/7, although the tubulin C-tails are absent from cryo-EM maps likely due to the proposed flexibility and disorder/order transition of the TTLL catalytic domains (*25*, *28*). In contrast, the TTLL11/MT interface is much simpler, comprising solely the MT-BHB (pink) and the “missing” active site interaction motif. In addition, the TTLL11 catalytic domain (magenta) orientation is opposite regarding the polarity of the MT (fig. S4). Given the pronounced selectivity of TTLL6 and TTLL7 toward the α- and β-tubulin C-tails, respectively, it seems reasonable to hypothesize that interaction interfaces in TTLL6/7/MT complexes, which are missing in TTLL11, contribute to the protomer selectivity of TTLL6/7.

The sequence homology between TTLL11 and other TTLL elongases is limited to the folded catalytic domain, which terminates at the residue S447 (fig. S2F). In TTLL6 and TTLL7, the catalytic domain is followed by a helix-loop-helix motif, designated MTBD, and the positively charged helices of MTBD have been shown to interact with MT protomers in the TTLL6/7 cryo-EM structures (fig. S4) (*25*, *28*). In TTLL11, the corresponding putative MTBD (residues 487 to 526) is absent from the TTLL11 cryo-EM map, suggesting it does not directly interact with the MT surface. The absence of MT interactions is further supported by the overall negatively charged electrostatic surface of the TTLL11 structural motif analogous to MTBDs of TTLL6/7 (fig. S4). Last, mutating the cationic patch of the putative MTBD-like motif (KKKR-EEEE mutant (K_488_KKR_491_ to E_488_EEE_491_) resulted in only a slight reduction in MT binding in our TIRF experiments (Fig. 3B). Together, the sequences corresponding to the MTBDs of TTLL6 and 7 do not necessarily constitute the obligatory MT-binding motif in other TTLL elongases and might be dispensable for their physiological functions as shown for TTLL11.

The unique mode of TTLL11 binding to MTs is further illustrated by the superposition of its MT-BHB and corresponding helix pairs of TTLL6/7 (fig. S15, A to C). In TTLL11, helices α_10_ and α_11_ of MT-BHB bind the MT protomer groove interface via a mixed ionic/hydrophobic mode, while the positively charged C-terminal end of helix α_9_ serves as the primary MT-interacting motif in TTLL6 (fig. S5, D and E) (*28*). These structural differences lead to distinct catalytic domain topologies: TTLL6/7 modify C-tails of tubulin protomers situated toward the plus end of the same PF, whereas TTLL11 glutamylates C-tails of protomers positioned toward the minus end of an adjacent PF (Fig. 2G and fig. S4). Moreover, the MT-BHB of TTLL11 does not show any structural homology with any other TTLL model (fig. S15D). Together, these findings suggest that TTLLs have evolved distinct substrate recognition strategies, albeit with some degree of cross-talk between the catalytic and MT-interacting domains. It would be interesting to explore in the future whether the paradigm of multipartite substrate recognition extends to other TTLL physiological substrates (*19*, *21*).

Our structural, biochemical, and functional data provide the following complex picture of TTLL11 preferences for polypeptides ending with glutamate (Fig. 6B). The primary driving force behind the elongation of the tubulin main chain is the peptide sequence and the biophysical characteristics of the C-terminal amino acid. It is evident that (poly)glutamate is preferred, although small aliphatic/hydrophobic residues are also tolerated, whereas bulky residues such as tyrosine are excluded (Figs. 5B and 6B). In addition, the isotype appears to be less critical, with the C-terminal tubulin sequence serving as the primary determinant of TTLL11 substrate specificity.

For both α- and β-tubulins, TTLL11 can salvage C-terminally truncated variants that were previously considered “dead-end sinks.” This generates unconventional polyglutamylation patterns within the tubulin code, directly linking polyglutamylation to the detyrosination/tyrosination cycle. First, as the αΔ2 variant could be reverted to αΔTyr, it can, in principle, serve as a reservoir for αΔTyr (or αTyr) tubulins in the cell. The spatiotemporal distribution, activity, and cross-talk between all key players of the detyrosination/tyrosination/polyglutamylation pathways are therefore of critical importance in regulating cytoskeleton-dependent cellular processes. For example, it can be assumed that in addition to detyrosination, C-terminal polyglutamylation may serve as yet another mechanism to regulate αTyr levels, thereby modulating processes mediated by αTyr readers, such as CAP-Gly proteins and kinesin-13 depolymerizing motors in the case of intracellular trafficking and MT disassembly, respectively (*39*–*43*). These findings highlight the potential of TTLL11 to contribute to the functional diversification of MT-related modifications within the tubulin code. However, further investigation is required to establish the physiological relevance of this mechanism.

Polyglutamylation of MTs is essential for regulating physiological processes such as intracellular transport, axonal growth, chromosomal separation, and ciliogenesis (*17*, *34*, *44*–*47*). As for the underlying molecular mechanisms, polyglutamylation profoundly affects the activity of enzymes that modify or interact with MTs. For example, MT-severing enzymes, such as spastin, preferentially bind polyglutamylated MTs (*22*), and polyglutamylation modulates the recruitment and activity of motor proteins such as dynein and kinesin. It also affects MT dynamics by controlling the activity of stabilizing or destabilizing MT-associated proteins, fine-tuning the structural and functional output of MTs in response to cellular demands (*32*, *48*–*51*). While polyglutamate chain length is known to affect these processes (*52*, *53*), it remains to be established whether tubulin code readers are sensitive to the nature of the linkage of glutamate chains, i.e., if they could distinguish between polyglutamylation at branching points and polyglutamylation extending tubulin primary chains. Overall, data reported here thus provide an impetus for more detailed biochemical and physiological studies focusing on these relationships.

### Limitations of the study

While our structural and biochemical findings provide strong evidence for TTLL11- and TTLL6-mediated elongation of tubulin primary chains, direct confirmation of this previously unrecognized polyglutamylation pattern in vivo remains technically challenging. This is due to the intrinsic complexity of tubulin C-terminal tails, which naturally contain stretches of glutamate residues, and the coexistence of overlapping PTMs. As a result, distinguishing between genetically encoded glutamates and those added posttranslationally requires the incorporation of isotopically labeled substrates—a strategy that is straightforward in vitro but extremely difficult to implement in a physiological context due to limited metabolic separation of translational and posttranslational glutamate pools. Although we attempted to model such conditions using ex vivo spliced mouse brain tissue in medium supplemented with labeled glutamate, its incorporation efficacy was insufficient for robust MS-based identification. Moreover, TTLL11 expression is very low in most commonly used cell lines, complicating genetic loss-of-function approaches. Consequently, our conclusions about TTLL11’s ability to elongate the main tubulin chain in vivo are currently based only on indirect evidence, including overexpression studies and substrate preferences observed in MS assays in vitro. Future development of tools to track posttranslationally added glutamates in living cells will be essential to definitively assess the physiological relevance of this modification.

## MATERIALS AND METHODS

### Sequences and cloning

Clones of hTTLL11 (UniProt, Q8NHH1), the inactive hTTLL11 (E441G) mutant, and murine CCP1 (mCCP1) were a kind gift of C. Janke (*18*). The TTL clone was reported previously (*3*). Truncated TTLL11 variants were polymerase chain reaction (PCR) amplified using corresponding sets of gene-specific primers (table S1) and cloned into the pDONR221 donor vector via the BP recombination reaction following the manufacturer’s protocol (BP Gateway cloning, New England Biolabs, MA, USA). Individual sequences were transferred into the pMM322 expression vector comprising the N-terminal Twin-Strep-FLAG-HALO-tag (*54*) using the LR recombination reaction. Sequence-optimized genes encoding human TMCP2 (Q8NCT3), ttTMCP (Q24D80), human TTLL6 (Q8N841), and human TTLL7 (Q6ZT98) were synthesized commercially (Thermo Fisher Scientific, MA, USA) and cloned into the pMM322 expression vectors as described above. The TTL gene was PCR amplified using corresponding sets of gene-specific primers (table S1) and cloned into the pEC566 expression vector containing the N-terminal His–maltose-binding protein (MBP) tag (*54*). All used plasmids are listed in table S2.

Vectors for lentiviral expression [pHR-CMV (expression), psPAX2 (packaging), and pMD2.G envelope] were provided by D. Rozbesky (*55*). The target genes were cloned into the pHR-CMV expression plasmid using the Gibson Assembly Master Mix (BioLabs Inc., New England) following the manufacturer’s protocol together with specific primers with overlapping target sequences (table S1). Nucleotide sequences of all plasmids were verified using Sanger sequencing. Site-directed mutagenesis was carried out by the standard QuikChange protocol (Agilent Technologies, CA, US) using matching pairs of complementary mutagenic primers (table S1).

### Protein expression and purification

#### Transduction of HEK293T cells

The published lentiviral transduction system was used to generate stable transformants of HEK293T cells (*55*). Briefly, 10 μg of pHR-CMV-TetO_2_ containing the gene of interest, 10 μg of psPAX2, and 10 μg of pMD2.G plasmid was mixed in the Dulbecco’s modified Eagle’s medium (DMEM)/F-12 serum free medium (SFM) in the total volume of 0.25 ml. Seventy-five micrograms of linear polyethyleneimine (lPEI; Polysciences Inc., Warrington, PA, USA) in 0.25 ml of DMEM/F-12/SFM was mixed with the plasmid solution and incubated at room temperature for 20 min. The mix was then added to a T75 flask with HEK293T Lenti-X cells at >90% confluency in 11.5 ml of fresh DMEM/F-12/2% fetal bovine serum (FBS) medium. The cells were incubated at 37°C and 5% CO_2_ for 72 hours. The conditioned medium was harvested, mixed with 6 ml of fresh DMEM/F-12/10% FBS medium, and filtered through a 0.45-μm filter unit. HEK293T cells (>90% confluency) were transduced with the medium containing the lentiviral particles in a T75 flask. The medium was exchanged after 3 days. To enrich positive cell population, the transduced cells were labeled with the 50 nM TAMRA-HALO probe for 30 min, the medium was replaced, and the cells were sorted using BD FACSAria Fusion (BD Biosciences, NJ, USA). Cells (100,000) were collected, transferred to DMEM/F-12/10% FBS medium, and expanded for ensuing experiments.

### Heterologous expression and purification

#### TTLL11 variants

hTTLL11 variants were expressed in suspension HEK293T cells as fusions with the N-terminal TwinStrep-Flag-HALO tag using established protocols (*54*, *56*). Briefly, a day before transfection, cells were seeded at the density of 2.0 × 10^6^ cells/ml in 350 ml of FreeStyle F17 medium (Thermo Fisher Scientific) supplemented with 2 mM l-glutamine and 0.1% Pluronic F-68 (Invitrogen) in 2-liter Erlenmeyer flasks. The cell suspension was incubated at 110 rpm under a humidified 5% CO_2_ atmosphere at 37°C overnight and the next day transfected with a mixture of 0.7 mg of an expression plasmid DNA diluted in 17.5 ml of phosphate-buffered saline (PBS) and 2.1 ml of lPEI (1 mg/ml). Four hours posttransfection, 350 ml of EX-CELL Serum-Free Medium (Merck Life Sciences, Darmstadt, Germany) was added to the cell suspension. Cells were harvested by centrifugation at 500*g*, 5 min, 4°C, and 72 hours posttransfection. The cell pellet was lysed in an ice-cold lysis buffer [100 mM tris-HCl (pH 8.0), 10 mM NaCl, 5 mM KCl, 2 mM MgCl_2_, 10% glycerol, 1 mM EDTA, 1 U/ml benzonase (Merck Life Sciences, Darmstadt, Germany), and 1× protease inhibitor cocktail (Roche, Basel, Switzerland)] by sonication (24 W/3 × 20 s/min). To assist cell lysis, IGEPAL 630 [0.2% (v/v)] was added to the cell lysate (20-min incubation on ice), followed by the addition of 4 M NaCl (150 mM final concentration). The cell lysate was then centrifuged sequentially at 9000*g* (15 min), 4°C, and 30,000 g (30 min) at 4°C.

For purification, the supernatant was mixed with a Strep-Tactin XT resin (IBA, Gottingen, Germany) equilibrated in 100 mM tris-HCl (pH 8.0), 150 mM NaCl, 5 mM KCl, 2 mM MgCl_2_, 10% glycerol, and 1 mM EDTA (equilibration buffer) and incubated at 4°C for 1 hour. The slurry was then loaded into a 10-ml plastic column and sequentially washed with the ice-cold equilibration buffer [20-column volumes (CVs)], 5 CVs of the equilibration buffer supplemented with 3 mM adenosine 5′-triphosphate (ATP) and 10 mM MgCl_2_, and lastly 5 CVs of the equilibration buffer. The fusion protein was eluted with 12 CVs of elution buffer [50 mM tris-HCl (pH 8.0), 150 mM NaCl, 10 mM KCl, 10% glycerol, 10 mM d-biotin, and 1 mM EDTA], and pooled elution fractions were concentrated to ~2 mg/ml, snap frozen in liquid nitrogen, and stored at −80°C. The sample was centrifuged at 20,000*g* at 4°C for 15 min, transferred onto a Nanosep 0.2-μm spin filter tube (Pall Corp., Port Washington, NY, USA), and centrifuged at 10,000*g* at 4°C for 5 min. The supernatant was injected onto a size-exclusion Superose 6 Increase 10/300 GL column (GE Healthcare Bio-Sciences Corp., Piscataway, NJ, USA) connected to the NGC Discover Pro System (Bio-Rad Laboratories, Hercules, CA, USA) equilibrated in the size exclusion chromatography (SEC) buffer [50 mM tris-HCl (pH 8), 140 mM NaCl, 10 mM KCl, 1 mM EDTA, 0.5 mM TCEP, and 5% glycerol]. Fractions comprising TTLL11 fusions were pooled, concentrated to ~1 mg/ml, snap frozen in liquid nitrogen, and stored at −80°C until further use. The protein purity was evaluated by SDS–polyacrylamide gel electrophoresis (SDS-PAGE) (Fig. 1C) with typical yields ranging from 150 to 800 μg/liter of cell culture.

#### Cytosolic carboxypeptidase 1

mCCP1 and the hTTLL11 (E531G) mutant were expressed in suspension stably transduced HEK293T cells as fusions with the N-terminal TwinStrep-Flag-HALO tag. Cells were grown in the 1:1 mixture of the FreeStyle F17 Expression Medium and the EX-CELL 293 Serum-Free Medium supplemented with 0.05% Pluronic F-68 and 2 mM l-glutamine at 110 rpm under a humidified 5% CO_2_ atmosphere at 37°C. Once reaching the density of 8.0 x 10^6^ cells/ml, cells were harvested by centrifugation at 500*g* at 4°C for 10 min, and recombinant proteins were purified by Strep-Tactin affinity chromatography as described above. For CCP1 purification, EDTA was omitted from all purification buffers.

#### Tubulin tyrosine ligase

TTL was expressed in BL21(DE3) RIPL *Escherichia coli* (Thermo Fisher Scientific) as a fusion with the N-terminal His-MBP tag. The cell culture was grown to an optical density (OD)_600_ = 0.8 at 37°C and then cooled to 18°C, expression was induced by the addition of 1 mM isopropyl-β-d-thiogalactopyranoside, and cells were cultivated overnight. Cells were harvested by centrifugation (10,000*g* at 4°C for 20 min), and the cell pellet was resuspended in buffer A (PBS supplemented with 10 mM MgCl_2_ and 1 mM TCEP) and the protease inhibitor cocktail (pH 7.5). Cells were lysed by three passes through EmulsiFlex (Avestin, Canada) reaching 110 bar and then centrifuged subsequently at 7000 and 30,000*g* at 4°C for 15 min each step. The fusion protein was purified from the cell supernatant by the Ni–nitrilotriacetic acid affinity chromatography using a HiTrap HP column (Cytiva, DE, USA) connected to FPLC NGC Discover 10 Pro. The system was equilibrated in the HiTrap (HT) buffer A, the supernatant was loaded, and the column was washed with the HT buffer A supplemented by 500 mM NaCl and fractions eluted by a gradient of imidazole (30 to 500 mM) in 50 mM tris-HCl, 10 mM MgCl_2_, 1 mM TCEP, and 10% glycerol (pH 7.5). Fractions containing the TTL fusion protein were pooled, concentrated, and diluted in 50 mM tris-HCl, 10 mM MgCl2, 1 mM TCEP, and 10% glycerol (pH 7.5) to lower the imidazole concentration to 10 mM. The final concentration of TTL was 18 mg/ml, and after flash freezing in liquid nitrogen, the protein was stored at −80°C until further use.

#### TTLL11 labeling for TIRF microscopy

The purified HALO-TTLL11 fusions were mixed with a threefold molar excess of the Janelia Fluor 549–HALO probe (5 mM stock solution in dimethyl sulfoxide; Promega) and incubated at 22°C for 30 min. The sample was centrifuged at 20,000*g* at 4°C for 15 min, transferred onto a Nanosep 0.2-μm spin filter tube (Pall Corp.), and centrifuged at 10,000*g* at 4°C for 5 min. The supernatant was injected onto a size-exclusion Superose 6 Increase 10/300 GL column (GE Healthcare Bio-Sciences Corp., Piscataway, NJ, USA) connected to the NGC Discover Pro System (Bio-Rad Laboratories) equilibrated in the SEC buffer [50 mM tris-HCl, 140 mM NaCl, 10 mM KCl, 1 mM EDTA, 0.5 mM TCEP, and 5% glycerol (pH 8)]. Fractions comprising Janelia Fluor 549–labeled TTLL11 fusions were pooled, concentrated to ~1 mg/ml, snap frozen in liquid nitrogen, and stored at −80°C until further use.

#### Purification of tubulin from HEK293T cells

Tubulin purification from suspension HEK293T was performed by two polymerization and depolymerization cycles based on a published protocol (*57*). Following the second depolymerization performed in BRB80, the protein was centrifuged, and the concentration was determined by absorbance measurement at 280 nm using a NanoDrop One Microvolume UV-Vis Spectrophotometer (Thermo Fisher Scientific). Purified tubulin was stored in aliquots (17 mg/ml) at −80°C.

#### Purification of porcine tubulin

The tubulin purification from pig brain followed a previously published protocol by polymerization/depolymerization cycles similar to that of tubulin from HEK293T cells (*58*). The concentration was determined by absorbance measurement at 280 nm using a NanoDrop One Microvolume UV-Vis Spectrophotometer (Thermo Scientific). The purified tubulin (20 mg/ml) was stored in aliquots in BRB80 buffer at −80°C.

### Preparation of α-tubulin variants

#### Tyrosinated tubulin

For tyrosination of tubulin isolated from HEK293T cells, a reaction mixture was prepared containing 50 μM tubulin, 4 μM TTL, 300 μM tyrosine, and 2 mM ATP in BRB80 [80 mM Pipes (pH 6.9), 1 mM EGTA, and 1 mM MgCl_2_]. The reaction was incubated at 22°C for 2 hours, followed by centrifugation at 20,000*g* for 10 min at room temperature. The resulting supernatant was aliquoted, snap frozen in liquid nitrogen, and stored at −80°C until further use.

#### αΔTyr, αΔ2, and αΔ3 tubulins

To prepare detyrosinated and Δ3 tubulin, a reaction mixture comprising 50 μM tubulin from HEK293T cells, 4 μM carboxypeptidase A (CPA; Merck Life Sciences, Darmstadt, Germany), and 3 μM recombinant mCCP1 (only in Δ3 tubulin preparations) in BRB30 [30 mM Pipes, 1 mM MgCl2, and 1 mM EGTA (pH 6.9)] supplemented with 1 μM ZnCl_2_ and 4 mM MgCl_2_ was incubated at 35°C for 1 hour. The reaction mixtures were then supplemented with an additional spike of equal dose of CPA (and mCCP1) and incubated for an additional 2 hours. To prepare polymerized MTs and remove mCCP1, reaction mixtures were supplemented with Pipes-Na at a final concentration of 330 mM [1 M stock solution (pH 6.9)] and 1 mM guanosine 5′-triphosphate and incubated at 37°C for 30 min. Polymerized MTs were centrifuged at 14,000*g* at 35°C for 30 min, resuspended in BRB80, aliquoted, snap frozen in liquid nitrogen, and stored at −80°C until further use.

#### Double stabilized MTs

Tubulin variants were polymerized as described previously (*59*). Briefly, tubulin aliquots stored at −80°C were thawed and centrifuged at 21,000*g* at 4°C for 10 min. The supernatant was transferred to a fresh Eppendorf tube, diluted (to 2.5 to 10 μM concentration) with the polymerization buffer [BRB80, 1 mM GMPCPP (Jena Bioscience, Jena, Germany), and 1 mM MgCl_2_], incubated on ice for 5 min, and then at 37°C for a minimum of 1 hour. The sample was centrifuged at 14,000*g* at 25°C for 30 min, the supernatant was carefully discarded, and pelleted MTs were dissolved in the BRB80 buffer supplemented with 10 μM Taxol to the final concentration of 10 μM. The dsMTs were stored at room temperature until further use.

### Tubulin glutamylation assay in vitro

Glutamylation reactions comprised 1 μM dsMTs and 0.3 μM TTLL11 variants in the BRB30 buffer supplemented with 1 mM TCEP, 2 mM ATP, 15 mM glutamate (l–glutamic acid monosodium salt monohydrate, Merck, Germany; L–glutamic acid 2,3,3,4,4-D_5_, 98%, Cambridge Isotope Laboratories, USA; or ^18^O-labeled glutamic acid synthesized in house), 4 mM MgCl_2_, and 0.5 mM EDTA. The mixture was incubated in an orbital shaker (300 rpm; Thermomixer Comfort, Eppendorf, Germany) at 25°C for a defined time in the range of 10 min to 2 hours. For the SDS-PAGE/Western blotting analyses, the reaction was stopped by the addition of the one-fifth volume of 5× SDS-PAGE sample buffer [250 mM tris-HCl (pH 6.8), 10% (w/v) SDS, 30% (v/v) glycerol, 5% (v/v) 2-mercaptoethanol, and 0.02% (w/v) bromphenol blue] supplemented with 250 mM dithiothreitol (DTT). For MS analyses, the reaction was centrifuged as described below.

### Tubulin glutamylation in cells

HEK293T cells were transfected with expression plasmids using the transfection reagent jetOPTIMUS (Polyplus, #101000025) according to the manufacturer’s instructions. Cells were lysed 24 hours after transfection by sonication in PBS. SDS-PAGE sample buffer (1×) and 200 mM DTT were added to the samples, and the samples were incubated at 60°C for 10 min, followed by heating at 95°C for 5 min. The samples were analyzed by SDS-PAGE and Western blotting.

### SDS-PAGE and Western blotting

α- and β-tubulins were resolved by SDS-PAGE composed of a 10% acrylamide separation gel [375 mM tris-HCl (pH 9), and 0.1% SDS (Sigma-Aldrich, #L5750)] and 3.5% stacking gel [125 mM tris-HCl (pH 6.8) and 0.1% SDS] prepared using the acrylamide/bis-acrylamide stock solution [40% acrylamide solution (Bio-Rad Laboratories, #161-0140) mixed with 0.54% (w/v) bis-acrylamide dry powder (Bio-Rad Laboratories, #161-0210)]. The running buffer was composed of 50 mM tris-HCl, 384 mM glycine, and 0.1% SDS (*60*). The separation was performed at 150 V for 80 min.

For immunoblotting, the proteins were transferred onto a polyvinylidene difluoride (PVDF) membrane using the Bio-Rad Trans-Blot Turbo Transfer System (Bio-Rad Laboratories) under standard conditions. The membrane was blocked with 5% (w/v) skimmed milk dissolved in tris-buffered saline (TBS). The membranes were probed with antibody solutions in 2.5% (w/v) skimmed milk in TBS (dilutions detailed in table S3) at 4°C for 8 and 2 hours for the primary and secondary antibodies, respectively. Secondary antibodies conjugated with horseradish peroxidase (HRP; Bio-Rad Laboratories) were used for the detection of chemiluminescence signals (Immobilon Forte Western HRP Substrate, Millipore, MA) and visualized using the ImageQuant LAS 4000 (GE Healthcare). The PageRuler Plus Prestained Ladder (Thermo Fisher Scientific) was used as a molecular weight marker.

### Mass spectrometry analysis of glutamylation

dsMTs from glutamylation assays were centrifuged at 14,000*g* at 22°C for 30 min. The resulting pellet, containing 26 μg of MTs, was resuspended in 5 μl of 50 mM ammonium bicarbonate (pH 7.8), supplemented with 100 mM DTT. Following incubation at 60°C for 30 min, freshly prepared 50 mM iodoacetamide (300 mM stock solution; 0.7 μl) was added, and the mixture was incubated in the dark for 30 min. Next, 150 mM DTT (1 M stock solution; 0.8 μl) and 0.65 μg (AspN; 2.5 μl) or 1 μg (trypsin/LysC mix; 1 μl) were added and incubated at 37°C (shaking at 650 rpm) overnight.

High-performance liquid chromatography (HPLC) separation was performed using an Agilent 1290 series HPLC system (Agilent Technologies). The sample (5 μl) was injected onto a reverse-phase trap column (Luna Omega Polar C18, 0.3 × 30 mm, Phenomenex, Torrance, CA), followed by a reverse-phase analytical column (Luna Omega Polar C18, 0.3 × 150 mm, Phenomenex), both heated to 50°C*.* Mobile phases were as follows: A [2% acetonitrile (ACN) and 0.1% formic acid (FA)] and B (98% ACN and 0.1% FA); the flow rate was 10 μl/min. The LC run consisted of a 35-min separation gradient of 5 to 40% B, a 30-min spike of 40 to 95% B, 3-min washing (95% B), a 1-min drop of 2 to 95% B, and equilibration of columns in 2% B for 10 min.

MS (quantification) and MS/MS (fragmentation) analyses were performed using a trapped ion mobility–quadrupole time-of-flight mass spectrometer (Bruker Daltonics, timsTOF Pro, Billerica, MA). Eluted peptides were analyzed by an MS acquisition method recording spectra from 250 to 2500 mass/charge ratio (*m/z*), and ion mobility was scanned from 0.6 to 1.73 Vs/cm^2^. For MS/MS, the protocol included a thermal ionization mass spectrometry survey scan of 100 ms, followed by 10 parallel accumulation serial fragmentation MS/MS scans, 150 ms for each of ion accumulation and ramp time. The total cycle time was 1.16 s. Target intensity was 20,000, the intensity threshold was 2500, and singly charged peptides with *m/z* < 800 were excluded by an inclusion/exclusion polygon filter applied within the ion mobility *m/z* heatmap. Precursors for data-dependent acquisition were fragmented with an ion mobility–dependent collision energy, which was linearly increased from 20 to 59 eV.

Intensities of peptides were determined using DataAnalysis 5.0 software (Bruker Daltonics). Visualization was performed in GraphPad Prism 8 (GraphPad Software, San Diego, CA, USA). Experimental data points represent mean values ± SD; *n* = 3. Fragmentation spectra were also analyzed in the same software, and fragment masses were determined using GPMAW 12.20 (General Protein/Mass Analysis for Windows) with the addition of the mass values of present isotopes (^13^C, D, and ^18^O). Masses of the labeled peptides selected for fragmentation and peptide envelope shapes were compared with theoretical spectra at https://envipat.eawag.ch. Because of the length of some analyzed peptides, fragments containing one or two ^13^C were used for the analysis in some cases. Fragments from b- and y-series were identified, showing the precise location of polyglutamate chains in each peptide.

### TIRF microscopy

TIRF microscopy in a combination with an interference reflection microscopy (*61*) was performed using an inverted microscope (Nikon-Ti E and Nikon-Ti2 E) [interference reflection microscopy (IRM); Nikon, Ti2-D-LHLED], equipped with a 100× numerical aperture (NA) 1.49 oil immersion objective (Nikon, SR Apo TIRF) and a PRIME BSI camera (Teledyne Photometrics, AZ, USA). Porcine dsMTs were visualized using the IRM, while Janelia Fluor 549–labeled TTLL11 variants were visualized with a 561-nm laser. Exposure time was 50 ms with the laser power set to 10%. The microscope was controlled using Nikon NIS-Elements software. All experiments were conducted at 22°C. Image analysis was carried out in the Fiji software (*62*).

Flow cells were prepared as described previously (*54*, *63*). Briefly, microscope chambers were built from silanized coverslips (Corning cover glass product) prepared as described previously (*64*). Parafilm was used to space two glasses and form channels of ∼0.1-mm thickness, 3-mm width, and 18-mm length. MTs were attached to the glass surface in each chamber via an anti–β-tubulin antibody (Sigma-Aldrich, T7816; 20 μg/ml in PBS). Subsequently, Janelia Fluor 549 TTLL11 variants were added to the chamber in the binding buffer [40 mM tris-HCl (pH 7.0), 1 mM TCEP, 1 mM MgCl_2_, and 5% (v/v) glycerol], casein (0.5 mg/ml), 10 μM paclitaxel, 0.001% C_12_E_8_ (dodecyloctaglycol), 20 mM d-glucose, glucose oxidase (110 μg/ml), and catalase (20 μg/ml). The fluorescent signal of Janelia Fluor 549–labeled TTLL11 variants was colocalized with the MTs viewed in the IRM channel. The signal intensity was quantified in Fiji by creating a linear region of interest (ROI) with a 3-pixel width along the MT. Then, the background with the same area near the MT was subtracted, and intensities per μm of MT were calculated. Intensities were normalized to the mean signal of the control and visualized using GraphPad Prism.

### Cryo-EM of the TTLL/MT complex

#### Sample preparation

dsMTs were centrifuged at 14,000*g* at 25°C for 30 min and resuspended to 50 μM concentration in the EM buffer [40 mM tris-HCl (pH 7.0), 1 mM TCEP, 1 mM MgCl_2_, and 5% glycerol]. The final mixture comprised 10 μM TTLL11 (E441G) and 10 μM dsMTs in the EM buffer. Cryo-EM grids Quantifoil R 2/1, Cu 300 mesh (Quantifoil, Germany) were glow discharged using a Leica EM ACE600 apparatus (Leica Microsystems, Germany) for 30 s. A Leica EM GP2 Automatic Plunge Freezer (Leica, Germany) was used with the following settings: 30-s wait time, 4-s blotting time, and automatic detection of the blotting force. Grids were first scanned using a JEOL JEM-2100Plus 200-kV microscope (JEOL, Germany), and the best grids were then used for data collection.

#### Data collection and processing

A total of 8800 movies were collected using Titan Krios (G1-2) (300 kV; Thermo Fisher Scientific) equipped with a Falcon 3EC direct electron camera (Thermo Fisher Scientific). The data were acquired with the following setup: 300 kV, cs = 2.7, apix = 1.349, frameDose = 1.0 e/A^2^ (30 frames), and nominal defocus range = −0.8 to −2.0 μm (fig. S3, A and B).

Data processing was carried out using the microtubule RELION-based pipeline (MiRP) (*65*). The 14-PF fraction of the data was used with the MiRP templates and assigned positions of homogenous segments by a series of MiRP three-dimensional (3D) classifications. Initial refinements were performed in RELION using a template map from prior 3D classification, the optimized reference map from the refinement, and, lastly, a helical symmetrization. As evaluating different refinement protocols for the whole MT map did not result in map improvements, symmetry expansion followed by particle subtraction was carried out for smaller segments of the map (from the 12 tubulin units down to an isolated TTLL11 molecule) to account for the presumed flexibility of TTLL11 at the MT surface. Different classifications were tested for each set of particles, and the refinement using multiple masks of different sizes and edge expansions was carried out both in RELION (*66*) and CryoSPARC (*67*). Final data processing steps were performed in CryoSPARC (*67*), and final refinements were done using a series of local searches exploiting masks containing either 12 or 4 decorated tubulin units. For postprocessing, we used DeepEMhancer with the highRes setting (*68*). Fourier shell correlation curves in CryoSPARC were used to estimate resolutions of each reconstruction using the gold standard. To estimate the resolutions for different parts of the map, a local resolution job was run in CryoSPARC. The tubulin part of the map has a maximum resolution of 2.7 Å, while the TTLL11 molecule is covered by a resolution gradient from 3.3 to 7 Å (Fig. 2C).

#### Model building

Initial atomic models were prepared in Coot adapted for cryo-EM (*69*) by mutating residues of existing GMPCPP-bound tubulin models [Protein Data Bank (PDB) ID: 6E7B] to match the human TUBB-5 and TUBA1B sequences. For TTLL11, an AlphaFold model of hTTLL11 was used. For atomic model building and refinement, a tubulin tetramer and TTLL11 were first docked into the density maps using rigid body fitting in ChimeraX (*70*). The structures were further refined using real space refinement in the Phenix suite (*71*), interspersed with the manual corrections to the model in Coot. The quality of the models was validated using the MolProbity server (*72*), and the model was deposited in the Research Collaboratory for Structural Bioinformatics under the accession number 9HQ4. Complete model statistics are reported in table S4.

### Assays on the SVBP KO mice brain and neurons

#### Animals

All experiments were conducted in accordance with the policy of the Grenoble Institute des Neurosciences (GIN). In compliance with the French legislation and European Union Directive of 22 September 2010 (2010/63/UE), the research was authorized by the Direction Départementale de la protection des populations–Prefecture de l’Isère-France and the ethics committee of GIN n° 004 accredited by the French Ministry of Research. SVBP-deleted mice (C57BL/6) were genotyped by PCR amplification (*35*). Primers for testing the SVBP mouse strain were 5′-GATCCACCTGCCCGGAAA-3′, 5′-TTTCTTCCAGCACCCTCTCC-3′, and 5′-CAAACCATGGATCCACGAAA-3′ as already described (*39*).

#### Brain protein extract preparation

Fifteen-week-old mice were euthanized by cervical dislocation. Cerebral hemispheres were extracted, rinsed in PBS solution, shock frozen in liquid nitrogen, and stored in liquid nitrogen until use. The organs (~200 mg) were lysed in 1-ml PEM buffer [100 mM Pipes, 1 mM EGTA, 1 mM MgCl_2_, and protease inhibitor cocktail (Roche Diagnostics, cOmplete Mini) at pH 6.7] using a Bead Mill 4 Thermo Fisher Scientific brand system with 1.4-mm ceramic beads. The lysate was centrifuged at 17,000*g* for 30 min at 4°C. The pellet was homogenized in 1-ml SDS buffer [3% SDS, 30 mM tris-base (pH 8.8), 5 mM EDTA, 30 mM NaF, 10% glycerol, and 1 mM DTT] and centrifuged at 17,000*g* for 15 min at 20°C. The supernatant was used for immunoblotting assays.

#### Neuronal cell culture and transfection

Mouse hippocampi (immunofluorescence) or cortices (immunoblot) were dissected from embryos (embryonic day 17.5) and digested in 0.25% trypsin in Hanks’ balanced salt solution (Invitrogen, France) at 37°C for 15 min. After manual dissociation, neurons were plated on poly-l-lysine–coated dishes (0.5 to 1 mg/ml), incubated for 2 hours in DMEM-10% horse serum, and then changed to magnetic cell sorting neuro medium (Miltenyi Biotec, Singapore) with B27 supplement (Invitrogen, France). Neurons were transfected using Amaxa Nucleofector kits (Lonza, Switzerland). At day 0 of culture, hippocampal cells from one or two embryos were used for a transfection point with 5 or 10 μg of plasmid, allowing expression of active or catalytically inactive TTLL11 together with cyan fluorescent protein (CFP) (kind gift of Janke lab, described in Lacroix *et al.* (*22*), and then cultured for 2 or 4 days. For immunoblotting, cortical cells cultured for 8 days in vitro were directly collected in the SDS-PAGE sample buffer.

#### Antibodies, immunoblotting, and immunofluorescence

Antibodies specific for total tubulin (mouse; α3A1 or β3A11), tyrosinated tubulin (rat; YL1/2), detyrosinated tubulin (rabbit), and Δ2-tubulin (rabbit) were described by Aillaud *et al.* (*11*), and chicken anti–green fluorescent protein (GFP) antibody was purchased from Sigma-Aldrich. A newly developed polyclonal antibody against poly-glutamate chain (anti–polyE Gre) was produced in a guinea pig using the peptide C-EEEEEEE linked at the N-terminal to the keyhole limpet hemocyanin protein via the cysteine. Validation of this antibody was performed in protein extracts from HEK293T cells overexpressing the TTLL enzyme. For immunoblotting, antibodies were all used at 1:10,000, except anti-polyE Gre, which was used at 1:5000. For immunofluorescence, antibodies were all used at 1:1000, except α3A1, which was used at 1:5000.

For immunoblotting, protein extracts were loaded on 10% acrylamide gels (Bio-Rad Laboratories, Mini-PROTEAN TGX Stain-Free) or homemade 10% acrylamide/bisacrylamide (37.5:1) for separation of α- and β-tubulin and transferred with Trans-Blot Turbo (Bio-Rad Laboratories) on PVDF membranes. Membranes were incubated with primary antibodies, with secondary fluorescent antibodies (antimouse conjugated to Alexa Fluor 488 and anti–guinea pig, antirabbit, and antirat conjugated to cyanine-3, all used at 1:2000) and last revealed with ChemiDoc camera (Bio-Rad Laboratories). The immunoreactive bands were visualized by ChemiDoc MP Imaging System (Bio-Rad Laboratories), and then the images were quantified and analyzed using Image Lab (Bio-Rad Laboratories) software. The quantifications were graphically plotted using GraphPad software.

For immunofluorescence, cells were fixed at 37°C in 4% paraformaldehyde, 4.2% sucrose, and PBS medium and permeabilized using 0.1% Triton X-100 and PBS. Cells were then incubated with primary antibodies, followed by incubation with antichicken conjugated to Alexa Fluor 488 (GFP), antimouse and anti–guinea pig conjugated to cyanine-3 (GT335 and PolyE Gre, respectively; fig. S16), or antirabbit conjugated to Alexa Fluor 647 (αΔ2), all at 1:500. Nuclei were stained using Hoescht 33,258 (1 μg/ml).

Transfected neurons were imaged on Axioscan (Zeiss, Germany) equipped with a 10× NA 0.45 objective. Several ROI were defined using QuPath (*73*) and processed in Fiji software. Background was subtracted, and cells were segmented after automatic thresholding on the average image of polyE- and Δ2-tubulin channels. After setting the background to “NaN,” the signal was quantified in CFP-positive and -negative neurons in the area defined by a circle (40 μm in diameter) centered at the cell nuclei. A two-way analysis of variance (ANOVA) followed by Šidák’s multiple comparison post test was used to evaluate the statistical significance of differences between experimental samples.

### Synthesis of ^18^O-labeled glutamate

All solvents used for synthesis were obtained from commercial sources. All chemicals were purchased from Sigma-Aldrich, Tokyo Chemical Industry Co., Combi-Blocks, or ARMAR isotopes (water-^18^O) and were used without further purification. Thin-layer chromatography was performed on Silica gel 60 F_254–_coated aluminum sheets (Merck Millipore). Products were purified by preparative scale HPLC on a JASCO PU-975 instrument (flow rate of 10 ml/min) equipped with a UV-975 ultraviolet (UV) detector and Waters YMC-Pack ODS-AM C18 Prep Column (5 μm and 20 mm by 250 mm). The purity of compounds was assessed on an analytical Jasco PU-1580 HPLC (flow rate of 1 ml/min; invariable gradient from 2 to 100% ACN in 30 min) with a Watrex C18 Analytical Column (5 μm and 250 mm by 5 mm). ^1^H and ^13^C nuclear magnetic resonance (NMR) spectra were measured using Bruker Avance III HD 400 MHz, Bruker Avance III HD 500 MHz, and Bruker Avance III 600 MHz instruments. The internal signal of tetramethylsilane (δ = 0.0; CDCl_3_), residual signal of CDCl_3_ (δ = 7.26), or D_2_O (δ = 4.79) was used for standardization of ^1^H NMR spectra. NMR spectra were recorded at room temperature unless noted otherwise. Chemical shifts are given in δ scale; coupling constants (*J*) are given in cycles per second. The electrospray ionization (ESI) mass spectra were recorded using a ZQ micromass mass spectrometer (Waters) equipped with an ESCi multimode ion source and controlled by MassLynx software. Low-resolution ESI mass spectra were recorded using a quadrupole orthogonal acceleration time-of-flight tandem mass spectrometer (Waters, Q-Tof micro) and high-resolution ESI mass spectra using a hybrid Fourier transform mass spectrometer combining a linear ion trap MS and Orbitrap mass analyzer (Thermo Fisher Scientific, LTQ Orbitrap XL). The conditions were optimized for suitable ionization in the ESI Orbitrap source [sheath gas flow rate of 35 arbitrary units (a.u.), auxiliary gas flow rate of 10 a.u. of nitrogen, source voltage of 4.3 kV, capillary voltage of 40 V, capillary temperature of 275°C, and tube lens voltage of 155 V]. The samples were dissolved in methanol and applied by direct injection.

Sodium metal (11.0 mg, 0.48 mmol, and 3.0 equiv.) was carefully dissolved in water-^18^O (0.1 ml) under an argon atmosphere at 0°C. This solution was added to (*S*)-2-((*tert*-butoxycarbonyl)amino)-5-methoxy-5-oxopentanoic acid (42 mg, 0.16 mmol, and 1.0 equiv.), and the resulting mixture was stirred under an argon atmosphere at room temperature for 1 hour. The reaction mixture was then frozen and lyophilized to afford 40 mg (quantitative yield) of (*tert*-butoxycarbonyl)-l-glutamic-γ-^18^O acid (fig. S17) as a white solid. This intermediate was used for the next step without further purification [ESI MS: 272.1 ([M + Na]^+^)].

(*tert*-Butoxycarbonyl)-l-glutamic-γ-^18^O acid (fig. S17, 40 mg, and 0.16 mmol) was dissolved in 4 M HCl/dioxane (0.2 ml), and the resulting mixture was stirred under an argon atmosphere at room temperature for 2 hours. The reaction mixture was then frozen and lyophilized to afford 29 mg (quantitative yield) of l-glutamic-γ-^18^O acid hydrochloride (fig. S17) as a white solid [^1^H NMR (401 MHz, D_2_O): δ 2.10 to 2.25 (m, 2H), 2.59 (td, *J* = 2.7, 7.8 Hz, 2H), 3.89 (t, *J* = 6.5 Hz, 1H); ESI MS: 148.0 ([M - H]^+^); HR ESI MS: calculated for C_5_H_8_O_3_^18^ON: 148.05013; found: 148.04988].

## Supplementary Materials

### This PDF file includes:

Figs. S1 to S17 Tables S1 to S4 References
