# Supplementary material for: Mechanistic insights into TTLL11 polyglutamylase–mediated primary tubulin chain elongation
Source: Sci Adv. 2025 Aug 20;11(34):eadw1561. doi: 10.1126/sciadv.adw1561 (PMC12366701; doi:10.1126/sciadv.adw1561)
Supplement: Supplementary file 1 — Figs. S1 to S17 Tables S1 to S4 References [file sciadv.adw1561_sm.pdf]

Science Advances
AAAS

# Supplementary Materials for

## Mechanistic insights into TTLL11 polyglutamylase–mediated primary tubulin chain elongation

Jana Campbell *et al.*

Corresponding author: Cyril Barinka, cyril.barinka@ibt.cas.cz

*Sci. Adv.* **11**, eadw1561 (2025)
DOI: 10.1126/sciadv.adw1561

**This PDF file includes:**

Figs. S1 to S17
Tables S1 to S4
References

**Fig. S1.**

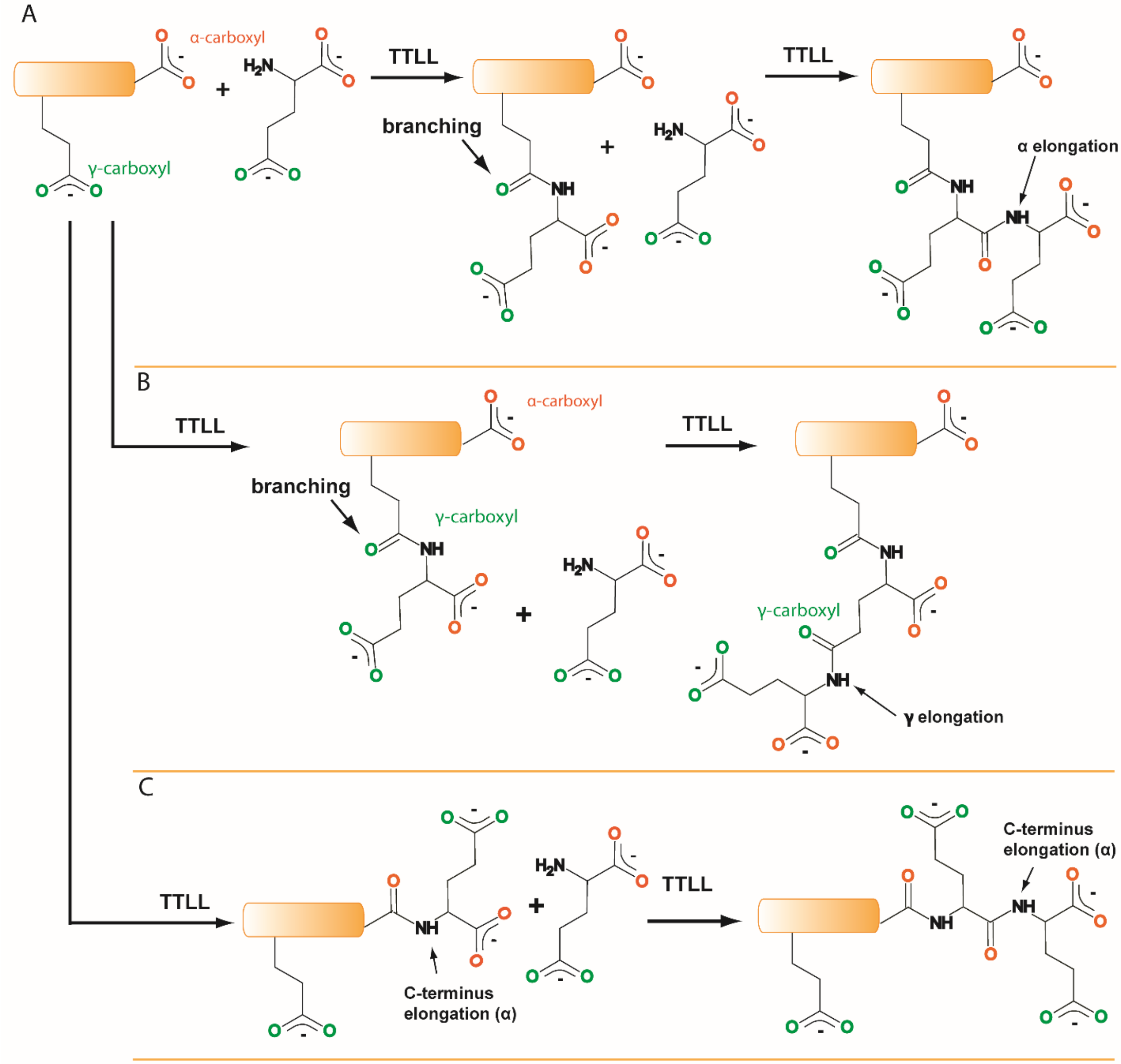


**Fig. S1. Theoretical polyglutamylation patterns.**
Multiple (theoretical) polyglutamylation patterns can co-exist in a target protein. **A. The current concept of protein polyglutamylation**. During the first initiation (branching) step, a glutamate residue is attached to the γ-carboxyl group of a glutamate side chain within the main peptide chain. The branching point is further extended by adding more glutamates via the α-carboxyl linkage. **B. An alternative polyglutamylation pathway.** The initiation step is followed by elongation steps that can attach glutamates via either of α- or γ-carboxylates of the growing polyglutamate chain. **C. A previously unrecognized polyglutamylation pattern reported here.** The first glutamate residue is attached to the free α-carboxylate of the terminal residue of the main polypeptide chain, resulting in direct extension of the protein sequence. In subsequent steps, the process is repeated as the additional glutamates are attached to the terminal α-carboxyl group.

**Fig. S2.**

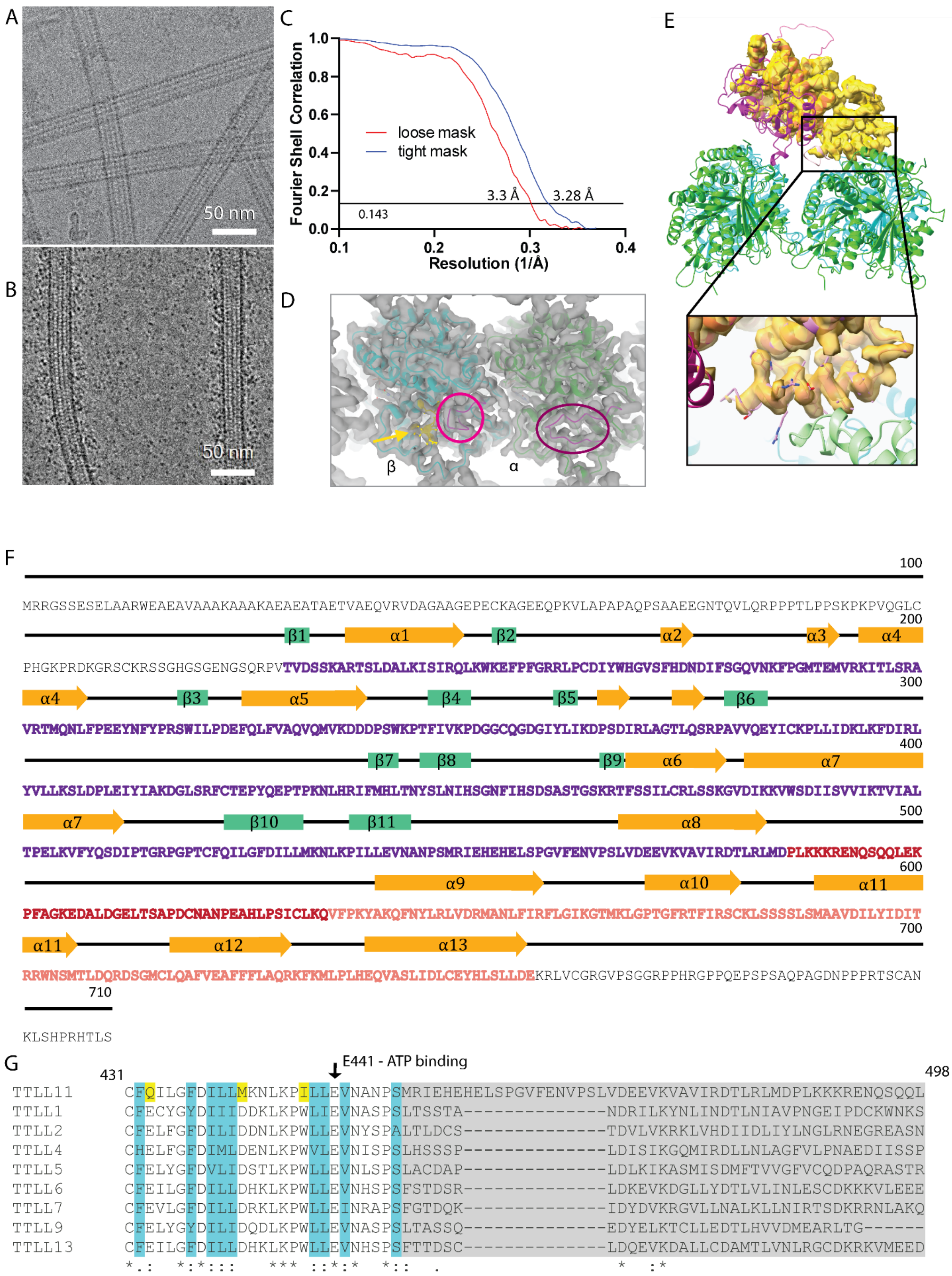


**Fig. S2. Structure of TTLL11 and its MT complex.**

**A,B. Cryo-EM image of MTs and MTs with bound TTLL11** (57 000x magnification), respectively. **C. The Fourier shell correlation curves of the cryo-EM map of the TTLL11/MT complex** from Cryosparc with tight and loose mask showing the overall resolution of the map is 3.28 Å. **D. The cryo-EM map (grey) allows for the unequivocal assignment of α and β-tubulin protomers**. The α-tubulin loop Y357-Q372 (purple oval), and β-tubulin loop D355-K362 (analogous, but 8 amino-acid shorter than in α-tubulin, hot pink oval), and the taxol-binding pocket of β-tubulin (yellow) are highlighted. This difference between the two isotypes is commonly used in cryo-EM to assign the protomers. **E.** Cryo-EM map of TTLL11 (gold) with fitted model of TTLL11 (catalytic domain-purple, MT-BHB-pink) with zoom in at α11 helix and its interface with tubulin (α-tubulin – green, β-tubulin – cyan). **F. The sequence and secondary structure prediction of human TTLL11.** The sequence is colored based on the tertiary structure model (black – intrinsically disordered N- and C-terminus, purple – the catalytic domain, dark red – the MTBD-like motif, pink – the MT-BHB) and the secondary structure elements are shown as yellow arrows and green bars for α-helices and β-sheets, respectively. **G. A partial sequence alignment of TTLL glutamylases**. The sequences spanning the C-terminal part of the catalytic domain (corresponding to residues C431 – S447) extending into the beginning of MT-binding motives were aligned using Clustal Omega. While there is high sequence conservation within the catalytic domains of all enzymes (blue – similar amino acids, yellow – conserved among TTLLs but different in TTLL11) there is a limited identity sequence beyond the catalytic domain in segments corresponding to the putative MT-binding motives.

**Fig. S3.**

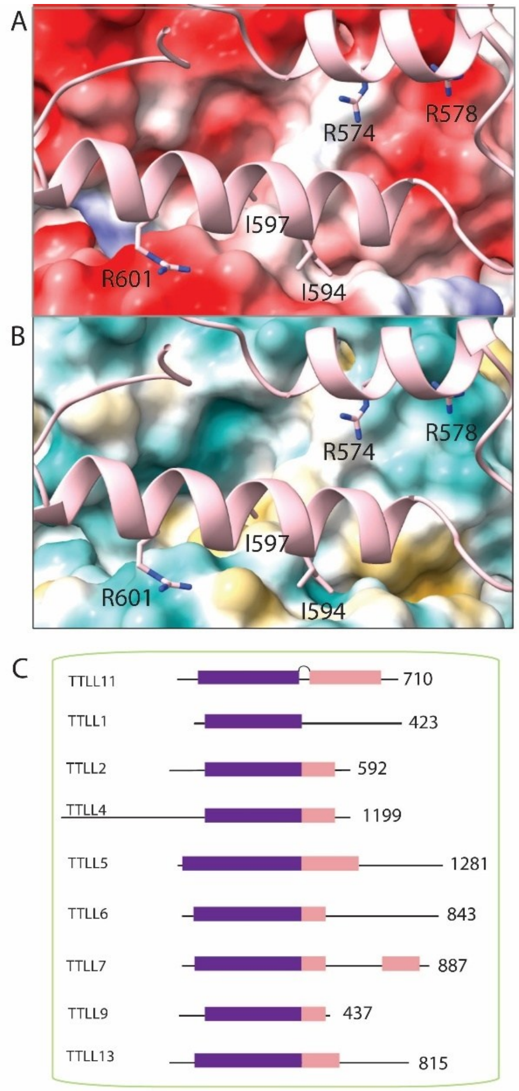


**Fig. S3. Interactions between TTLL11 and MT.**

**A,B. Interactions between MT-BHB of TTLL11 and the MT surface.** The α10 and α11 helices of TTLL11 (shown in cartoon representation, pink) represent the primary TTLL11/MT interaction motif. The interaction interface includes **(A)** ionic interactions between the negatively charged MT surface (ChimeraX, colored by Coulombic electrostatic potential red – electronegative, blue – electropositive) and positively charged side chains of R574, R578, and R601; (**B**) hydrophobic interactions between I594 and primarily I597 of TTLL11 and hydrophobic patches at the MT surface (ChimeraX, colored by molecular lipophilic potential green – lipophobic value -20, yellow – lipophilic value 20). **C**. **Schematic representation of TTLL glutamylases**. All enzymes have a high degree of similarity between their catalytic domains (purple). The C-terminal domains usually involved in interactions with MTs (MT-binding domains; pink) vary substantially both in length and sequence. Intrinsically disordered N- and C-termini are shown as black lines.

**Fig. S4.**

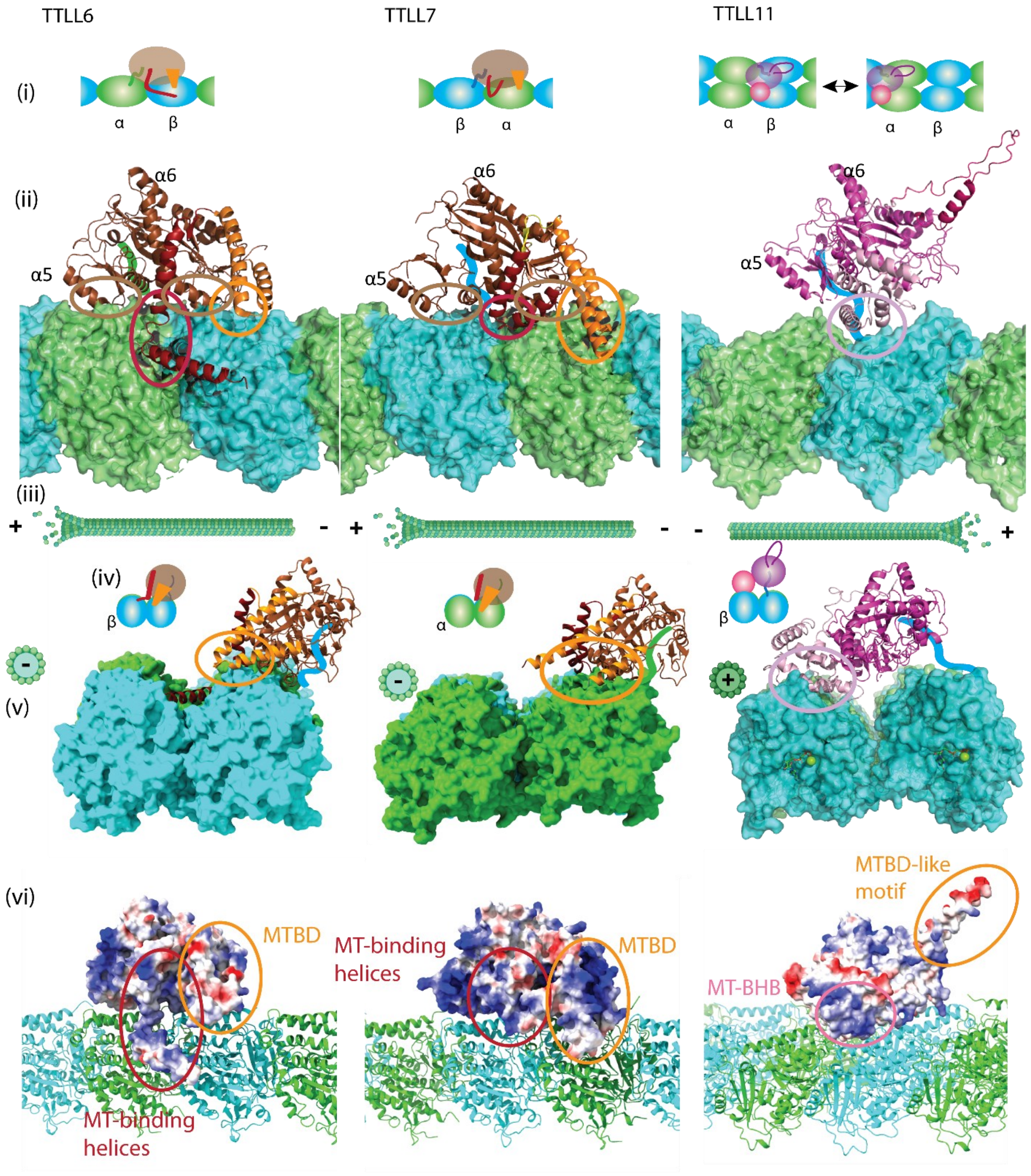


**Fig. S4. Comparison of cryo-EM structures of the TTLL6/7/11 complexes with MTs.**

Structural models and schematic representations of TTLL/MT complexes (front - ii and side view - v) are derived from the structures of TTLL6, TTLL7, and TTLL11/MT complexes (8U3Z (28), provided by Antonina Roll-Mecak (25) and 9HQ4, respectively). Tubulin protomers are shown in surface representation and colored green and cyan for α and β-tubulin, respectively, C-tails that protrude into the active sites of TTLLs are shown as wavy lines of corresponding colors. TTLLs are shown as cartoons and for TTLLs 6/7 the coloring is: the catalytic domain – brown, MTBD – orange, and the MT binding helices – dark red. For TTLL11: the catalytic domain – magenta, MTBD-like motif – dark red, the MT-BHB – pink. Interaction interfaces are highlighted by ovals of matching colors. For easier orientation in the positioning of the protein structures, schematic representation of both orientations was added (i, iv). In addition to TTLL interactions with the tubulin C-tails extended to their active sites, TTLL6/7 engage MTs by three additional interfaces, while TTLL11/MT interaction interface comprise only helices α10 and α11 of the MT-BHB. The complex

interaction interfaces of TTLL6/7/MT, absent in TTLL11, can contribute to the protomer selectivity of the enzymes. The positioning of the catalytic domains in relation to MT differs (iii). In the bottom part (vi), TTLL6/7/11 surfaces are colored by Coulombic electrostatic potential (ChimeraX, red – electronegative, blue – electropositive), tubulins are shown in the cartoon representation. As expected, MT-binding helices as well as MTBD of TTLL7 are positively charged to facilitate interactions with the negatively charged surface of MTs. In the case of TTLL11, MT-BHB is also positively charged while the MTBD-like segment is negatively charged and thus less likely to interact with the MT surface (as also corroborated by our microscopic data).

**Fig. S5.**

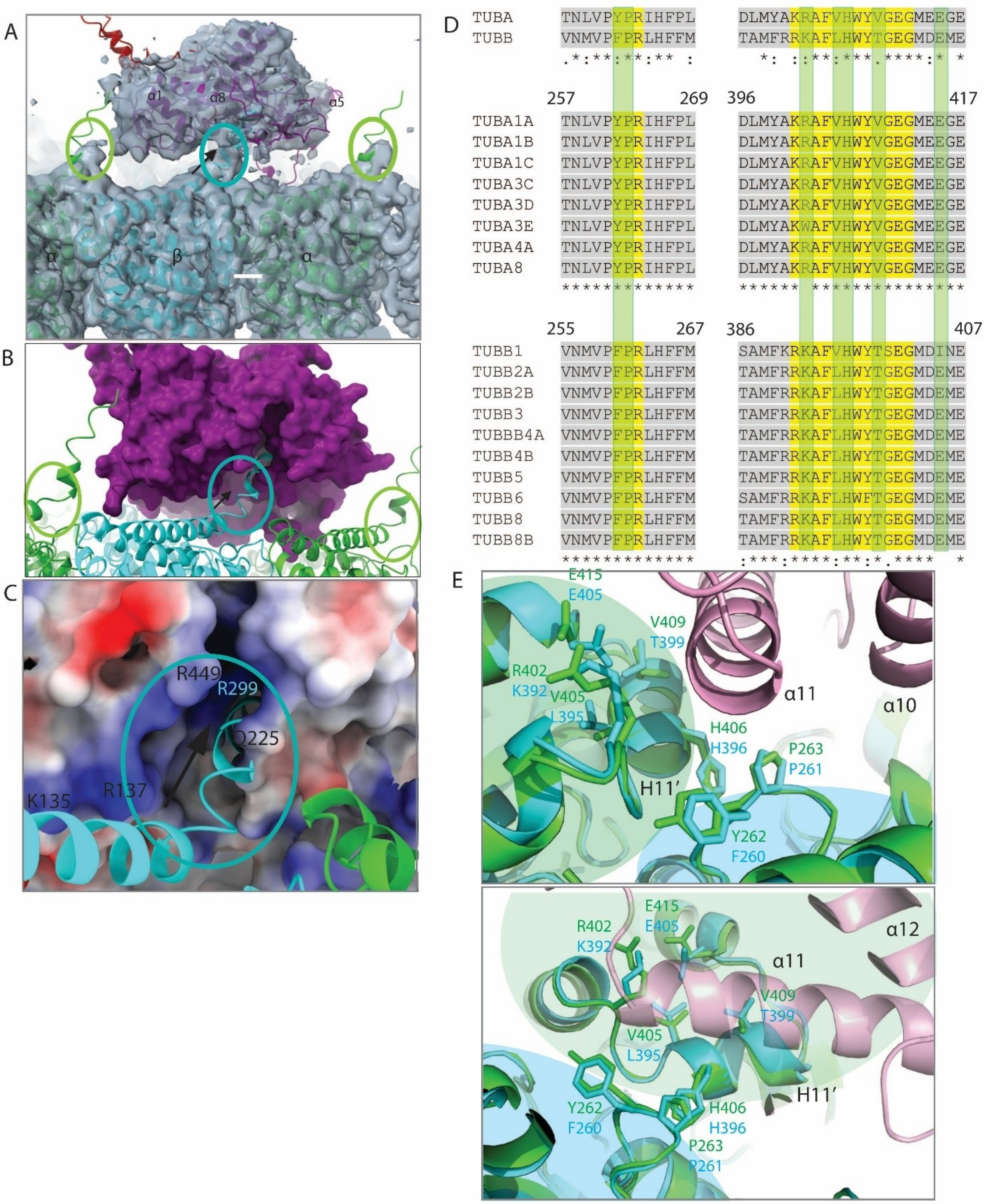


**Fig. S5. The position of tubulin tails is the TTLL11/MT complex and tubulin sequence conservation at intra- and inter-dimer longitudinal interfaces.**

**A-C. The active site of TTLL11 accommodates the C-tail of β-tubulin.** The TTLL11/MT complex model fitted in the cryo-EM map with a low contour level (0.15). Proteins are shown in cartoon representation and colored green, cyan, magenta/red/pink for α-tubulin, β-tubulin, the TTLL11, respectively. Given their inherent flexibility, only N-terminal segments of tubulin C-tails are visible in the cryo-EM map (green and cyan ovals). Tubulin comprising the "full-length C-tails" were modeled in AlphaFold and manually inserted into the map. The C-terminus of β-tubulin extends into the active site tunnel of TTLL11 (black arrow). **B**. The identical model as in **A**, with TTLL11 shown in surface representation to better visualize the TTLL11 active site tunnel. **C**. TTLL11 shown in surface representation colored

by the Coulombic electrostatic potential (ChimeraX, red – electronegative, blue – electropositive) showing the positive charge of the tunnel and its immediate surroundings attracting the negatively charged tubulin tails. **D. Sequences of human tubulin isoforms were aligned using Clustal Omega**. Sequences at intra- and interdimer groove interfaces are marked yellow, while residues involved in interactions with TTLL11 are highlighted by green boxes. **E**. **Structural superposition of intra- and interdimer groove interfaces.** 3D structures of tubulin isoforms TUBA1B and TUBB5 (42 % identity, 61 % overall similarity) were superimposed on the corresponding Cα atoms. Given the sequence and structural conservation at both intra- and interdimer groove interfaces, TTLL11 cannot effectively discriminate between them. α and β-tubulin are colored green and cyan, respectively, with amino acids labeled in corresponding colors. The green/cyan background highlight the original tubulin isotype in the cryo-EM structure – upper panel (MT-BHB front view), lower panel (MT-BHB left side view). TTLL11 is colored pink (semitransparent).

**Fig. S6.**

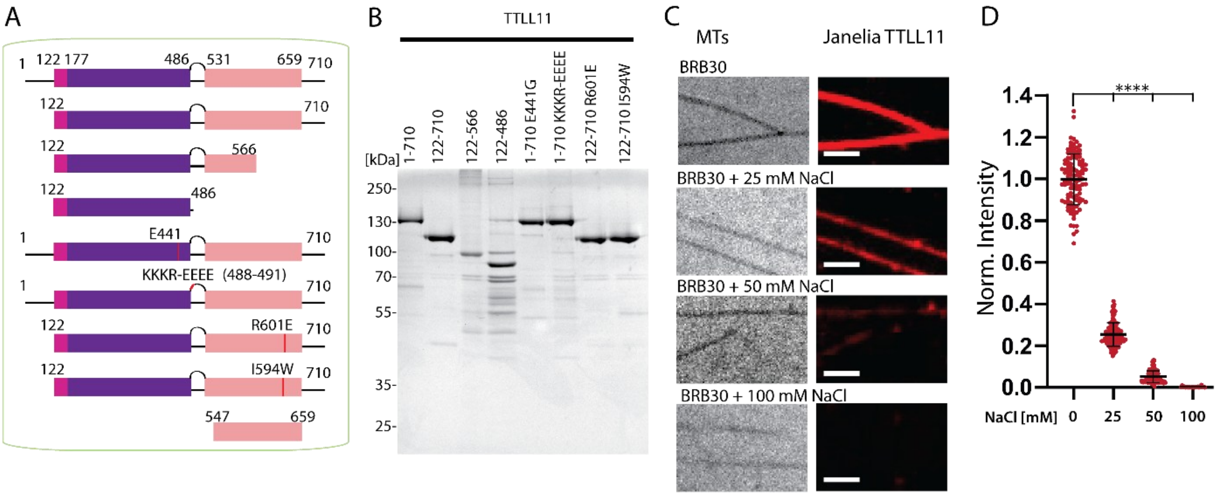


**Fig. S6. TTLL11 variants and TTLL11/MT binding dependance on ionic strength.**

**A. Schematic representation of TTLL11 variants used in this study.** The catalytic domain, the N-terminal extension, and the MT-BHB are colored purple, hot pink, and pink, respectively. Intrinsically disordered N- and C-termini are shown as black lines, the MTBD-like motif is shown as the black loop. Site-directed mutants are marked by the red line. E441G is a mutation in the catalytic site, KKKR-EEEE is a mutation in the MTBD-like motif changing the charge of the patch from positive to negative, R601E and I594W are mutations in the MT-BHB/MT interface. **B. The CCB-stained gel showing purified TTLL11 variants** comprising the N-terminal TwinStrep-FLAG-HALO tag. Full-length TTLL11 protein (1-710) as well as several truncated variants, designed based on structural predictions and the TTLL11/MT cryo-EM structure, were heterologously expressed in HEK293T cells and purified by the combination of affinity and SEC chromatography. The E531G represents an inactive mutant, KKKR-EEEE ($K_{488}KKR_{491}$-$E_{488}EEE_{491}$) is a mutation of a positively charged motif of MTBD-like motif (originally predicted to be involved in MT binding) (30), and the R691E and I684W are mutations in the MT binding helix α11 of MT-BHB. **C. Binding of TTLL11 to MT surface depends on salt concentration evaluated by TIRF microscopy.** MTs (black) were attached to the glass surface and the binding of TTLL11 (100 nM, conjugated to Janelia-549 fluorophore, red) visualized TIRF microscopy in a buffer comprising 40 mM Tris-HCl, pH7, 5 % glycerol, 1mM TCEP, 1mM $MgCl_2$ and varied concentrations of NaCl (0 – 100 mM). The TTLL11 binding intensity to MTs correlated negatively with an increase of the ionic strength of the assay buffer. Scale bar = 2 µm. **D. Quantification of TIRF images.** The fluorescence intensity of the Janelia-TTLL11 with subtracted background was normalized to MT length and compared the original conditions with added salt concentrations. 50 mM salt decreases the binding almost to the level of background signal. Data is shown as mean fluorescent intensity with $n = 2$ replicates, with 109, 98, 118, 35 MTs quantified in each sample. Statistical significance was determined using the unpaired t-test with Welsch correction, ****$p$<0.0001, the black bar represents median value with 95 % c.i..

**Fig. S7.**

A

| Tubulin variant | Enzymes used for preparation | C-terminal sequence |
|---|---|---|
| αTyr | TTL | R-VDSVEGEGEEEGEEY |
| αΔTyr | CPA | R-VDSVEGEGEEEGEE |
| αΔ2 | CPA, CCP1 | R-VDSVEGEGEEEGE |
| αΔ3 | CPA, CCP1 | R-VDSVEGEGEEEG |
| β(5) | | R-TAEEEEDFGEEAEEEA |
| β(4B) | | R-TAEEEGEFEEEAEEEVA |
| β(5)Δ2 | ttTMCP | R-TAEEEEDFGEEAEE |
| β(5)Δ3 | ttTMCP | R-TAEEEEDFGEEAE |

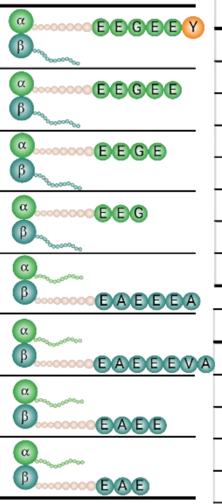

B

| α-tubulin | Norm. mRNA | C-terminal sequence |
|---|---|---|
| TUBA8 | 0.06 | R-TDSFEEENEGEEF |
| TUBA3E | 0.00 | R-VDSVEAEAEEGEAY |
| TUBA3C | 0.00 | R-VDSVEAEAEEGEEY |
| TUBA3D | 0.03 | R-VDSVEAEAEEGEEY |
| TUBA4A | 0.02 | R-IDSYEDEDEGEE |
| **TUBA1A - αTyr** | 5.13 | R-VDSVEGEGEEEGEEY |
| **TUBA1B - αTyr** | 78.08 | R-VDSVEGEGEEEGEEY |
| TUBA1C | 16.69 | R-ADSADGEDEGEEY |

| β-tubulin | Norm. mRNA | C-terminal sequence |
|---|---|---|
| TUBB1 | 0.01 | R-KAVLEEDEEVTEEAEMEPEDKGH |
| TUBB8 | 0.01 | R-TAEEEEDEEYAEEEVA |
| TUBB8B | 0.00 | R-TAEEEEDEEYAEEEVA |
| TUBB6 | 2.29 | R-TANDGEEAFEDEEEEIDG |
| TUBB3 | 0.77 | R-TAEEEGEMYEDDEEESEAQGPK |
| TUBB2A | 2.87 | R-TADEQGEFEEEEGEDEA |
| TUBB2B | 4.90 | R-TADEQGEFEEEEGEDEA |
| **TUBB5 - β(5)** | 65.80 | R-TAEEEEDFGEEAEEEA |
| TUBB4A | 0.54 | R-TAEEGEFEEEAEEEVA |
| **TUBB4B - β(4B)** | 22.82 | R-TAEEEGEFEEEAEEEVA |

**Fig. S7. Tubulin isoforms and C-terminal modification used in the study.**
**A**. **A list of tubulin variants used in this study.** Tubulin isoforms isolated from HEK293T cells primarily comprise isoforms TUBA1A/B, TUBB4B and TUBB5 (our LC-MS data). To enrich individual physiological tubulin variants differing in their C-terminal sequences, which can be underrepresented in HEK293T tubulin, purified tubulins were treated with recombinant tubulin-modifying enzymes (TTL, CPA, CCP1, and ttTMCP) or combinations thereof. **B**. **A list of human α- and β-tubulin isotypes in HEK293T cells** together with normalized mRNA intensities (30) and their corresponding C-terminal sequences. In line with our MS quantification, the most abundant isotypes include TUBA1A/B, TUBB4B and TUBB5. The predominant isoforms that were analyzed by MS in this study are in bold with added acronyms representing the C-terminal sequence.

**Fig. S8.**

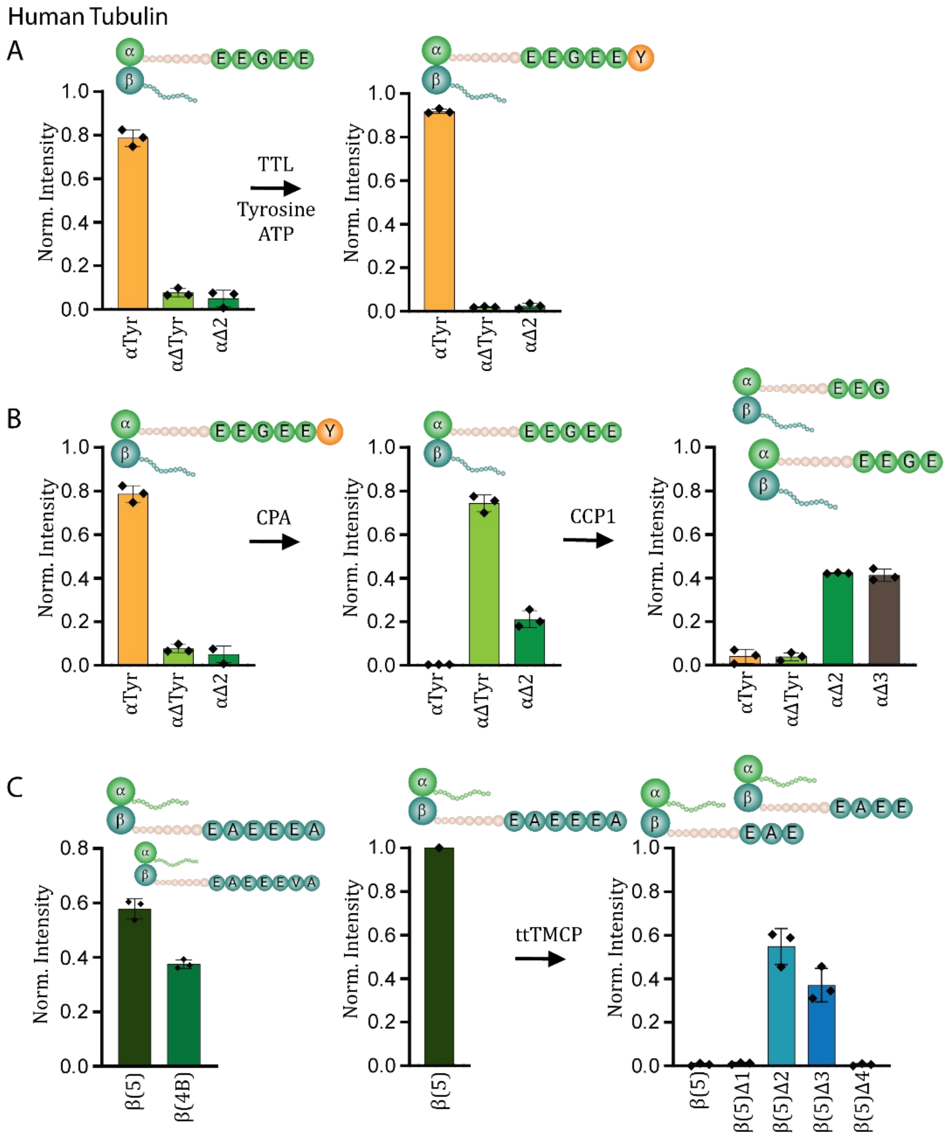


**Fig. S8. LC-MS based quantification of tubulin variants used in this study.**
**A**. **α-tubulin tyrosination.** αTyr represents the most abundant C-terminal variant of α-tubulins isolated from HEK293T cells, accounting for approximately 80% species, with remains of variants αΔTyr and αΔ2. TTL treatment was used to increase the abundance of the αTyr variant to over 90 %. **B. Generating αΔTyr, αΔ2, and αΔ3 enriched tubulins.** α-tubulins isolated from HEK293T cells contain only approximately 10 % of αΔTyr, while the αΔ3 variant was not detected. CPA treatment was used remove the C-terminal tyrosine to enrich the αΔTyr variant and the subsequent CCP1 treatment to further truncate the C-terminus to enrich the αΔ2 and αΔ3 fractions. **C**. **Generating β(5)Δ2 and β(5)Δ3 enriched tubulins.** β(5) and β(4B) represent the most abundant β-tubulin isotypes isolated from HEK293T cells. As the β(5) isoform is more abundant we focus our attention on this isoform, although both variants were present in reaction mixtures at the same time. β(5) was further modified by the treatment with recombinant TMCP resulting in tubulin fractions enriched in β(5)Δ2 and β(5)Δ3.

**Fig. S9.**

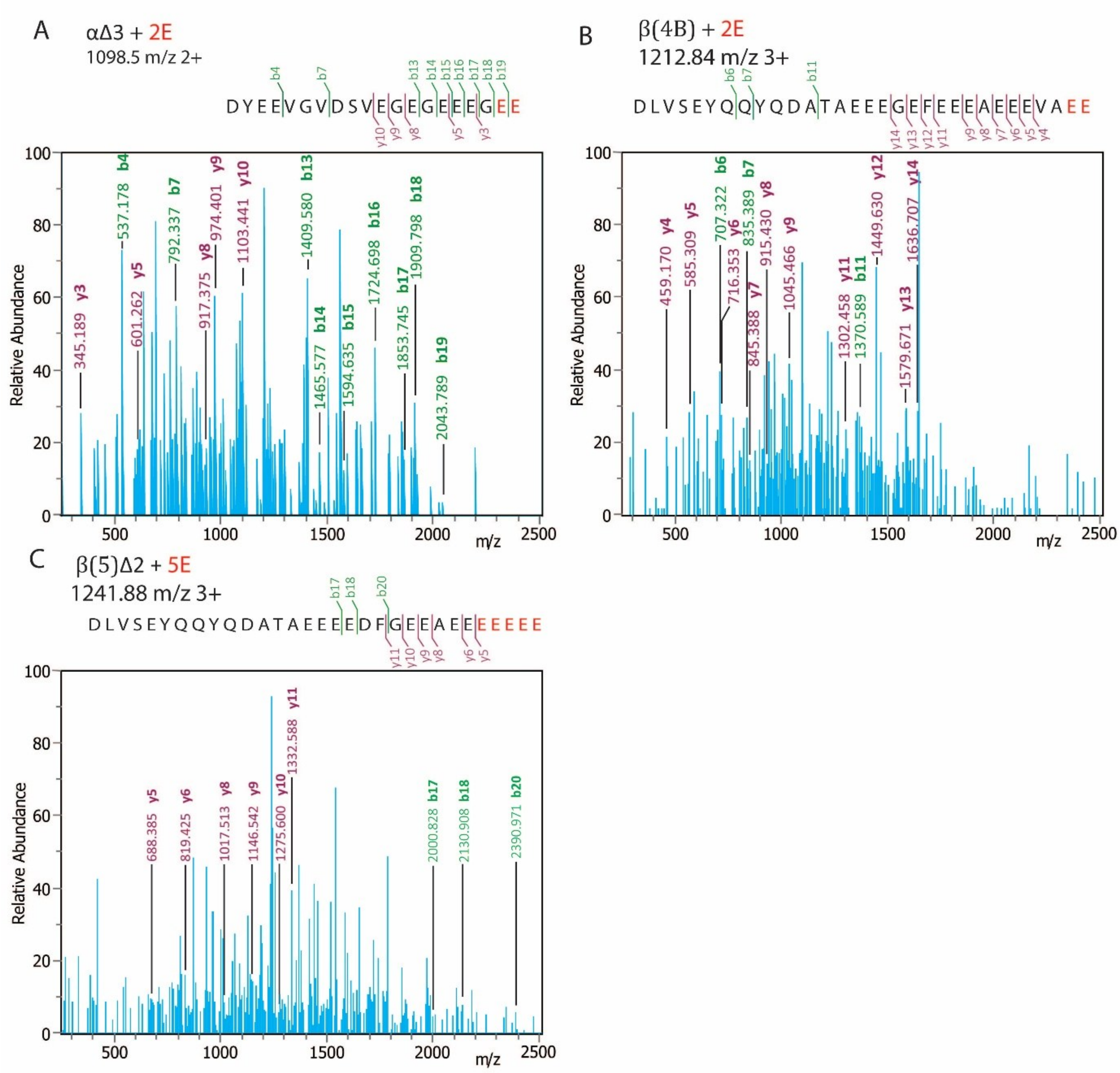


**Fig. S9. The LC-MS/MS analysis of tubulin glutamylation by TTLL11.**

**A-C. MS/MS spectra of polyglutamylated tubulin species.** The LC-MS/MS pipeline was used to analyze tubulin variants that were polyglutamylated by TTLL11 in the presence of isotopically labeled glutamate ($D_5$). Peaks of interest observed in b- and y-series of the fragmentation spectrum are highlighted in green and magenta, respectively. This approach enabled the unequivocal assignment of polyE attachment sites within the tubulin sequence. **A**. The αΔ3 peptide with 2 added isotopically labeled glutamates attached directly to the C-terminal glycine. **B**. The β(4B) peptide with 2 added isotopically labeled glutamates attached to the C-terminal alanine. **C**. The β(5)Δ2 peptide with 5 added isotopically labeled glutamates attached to the C-terminal glutamate.

**Fig. S10.**

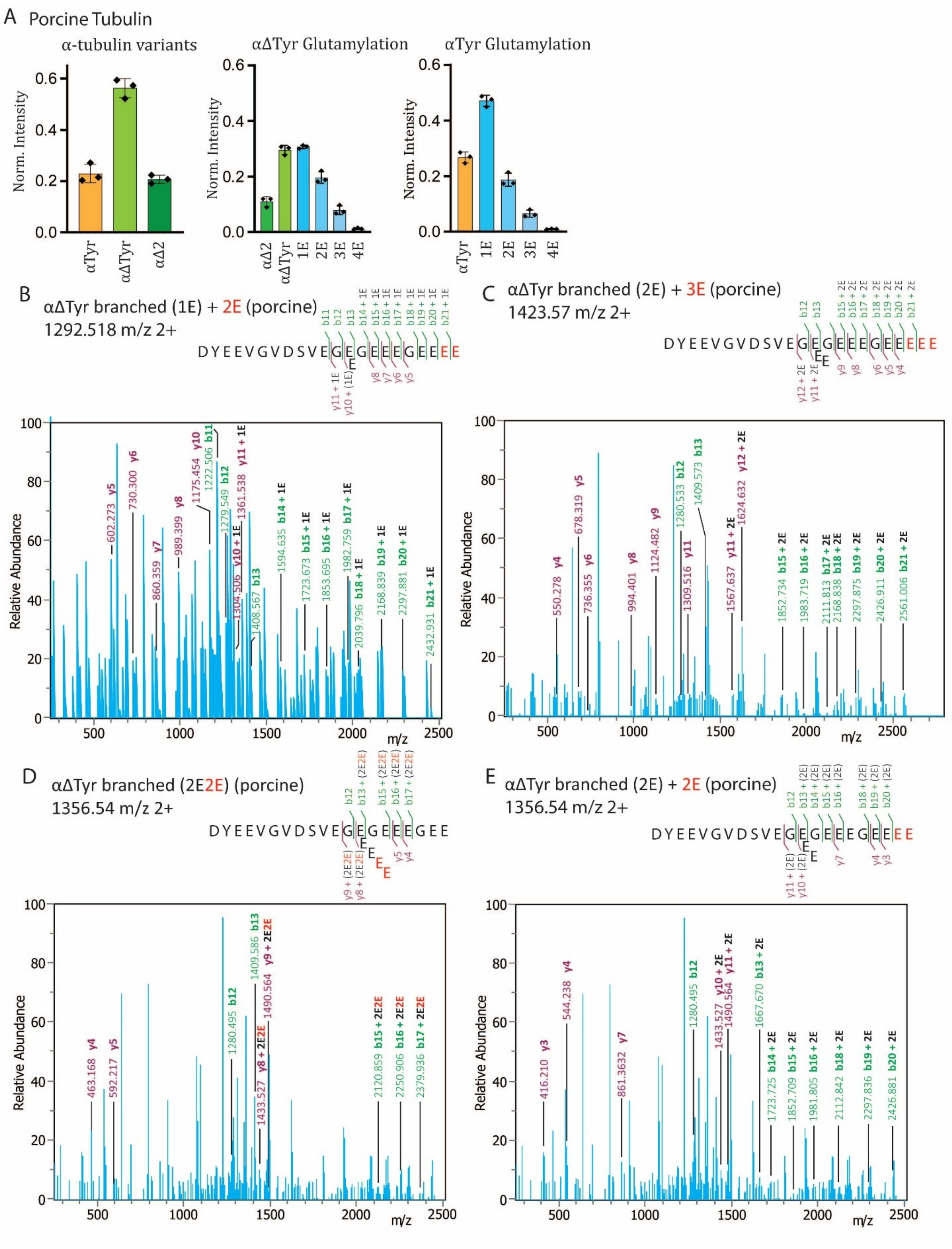


**Fig. S10. Quantitative and qualitative analysis of tubulin from porcine brain.**

**A. Quantification of tubulin isolated from porcine brains**. Porcine tubulins were isolated by polymerization/depolymerization cycles and individual variants quantitated by LC-MS. Compared to tubulin isolated from HEK293T cells, native porcine brain tubulins are extensively polyglutamylated. **B-E. MS/MS spectra of polyglutamylated tubulin species.** The LC-MS/MS pipeline was used to analyze tubulin variants that were

polyglutamylated by TTLL11 in the presence of isotopically labeled glutamate ($D_5$). Peaks of interest observed in b- and y-series of the fragmentation spectrum are highlighted in green and magenta, respectively. This approach enabled the unequivocal assignment of polyE attachment sites within the tubulin sequence. **B**. The αΔTyr branched (one glutamate at E443) peptide from porcine tubulin with 2 added isotopically labeled glutamates attached directly to the C-terminal glutamate. **C**. The αΔTyr branched (two glutamates at E443) peptide from porcine tubulin with 3 added isotopically labeled glutamates attached directly to the C-terminal glutamate. **D,E**. The αΔTyr branched (2 glutamates at E443) peptide from porcine tubulin with two added isotopically labeled glutamates attached to the glutamate branch (D) and at the same time to the C-terminus (E) as the m/z of these two variants is identical, they both come from the same peak in the MS spectrum and are only distinguishable in the MS/MS spectra.

**Fig. S11.**

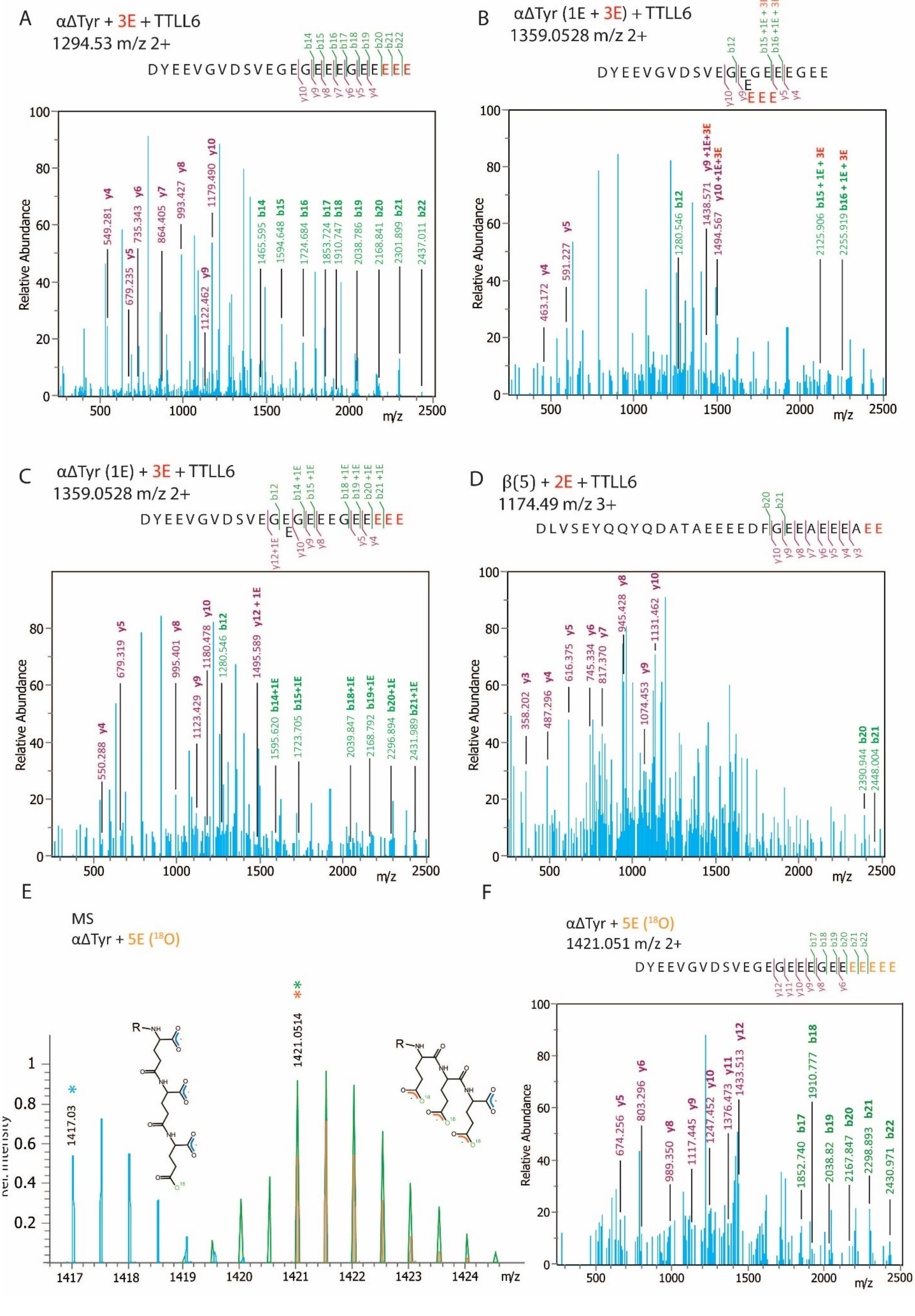

A
αΔTyr + 3E + TTLL6
1294.53 m/z 2+
DYEEVGVDSVEGEGEEEGEEEEE
B
αΔTyr (1E + 3E) + TTLL6
1359.0528 m/z 2+
DYEEVGVDSVEGEGEEEGEE
C
αΔTyr (1E) + 3E + TTLL6
1359.0528 m/z 2+
DYEEVGVDSVEGEGEEEGEEEEE
D
β(5) + 2E + TTLL6
1174.49 m/z 3+
DLVSEYQQYQDATAEEEEDFGEEAEEEAEE
E
MS
αΔTyr + 5E (18O)
1421.0514
1417.03
F
αΔTyr + 5E (18O)
1421.051 m/z 2+
DYEEVGVDSVEGEGEEEGEEEEEE
Relative Abundance
m/z

**Fig. S11. The LC-MS/MS analysis of tubulin glutamylation by TTLL6 and identification of the linkage of the TTLL11 polyglutamate chains.**

**A-D. MS/MS spectra of polyglutamylated tubulin species.** The LC-MS/MS pipeline was used to analyze tubulin variants that were polyglutamylated by TTLL6 in the presence of isotopically labeled glutamate ($D_5$). Peaks of interest observed in b- and y-series of the fragmentation spectrum are highlighted in green and magenta, respectively. This approach enabled the unequivocal assignment of polyE attachment sites within the tubulin sequence. **A**. The αΔTyr peptide with 3 added isotopically labeled glutamates attached to the C-terminal glutamate by TTLL6 showing that this type modification is not limited to only TTLL11. **B,C**. The αΔTyr peptide branched at E443 was glutamylated by TTLL6 both at the C-terminus (**C**) and the branch (**B**). **D**. The β(5) peptide with 2 added isotopically labeled glutamates attached to the C-terminal alanine by TTLL6. **E. Theoretical and experimental m/z spectra with $^{18}O$ labeled glutamylation.** MTs were incubated with TTLL11 in the presence of isotopically labeled glutamate $^{18}O$ located at the γ-carboxyl group and the spectrum of the αΔTyr C-terminal peptide extended by five glutamate units analyzed by LC-MS. Theoretical m/z spectra of the αΔTyr-EEEEE peptide with α- and γ-glutamate linkage within the pentaglutamate chain are colored orange and blue, respectively. The experimental m/z spectrum of the peptide (green) overlaps with the orange theoretical spectrum revealing the presence of the α-linkage within the pentaglutamate chain added by TTLL11 as no γ-carboxyl oxygens were released by creation of isopeptide bond. **F. The MS/MS fragmentation spectrum of the αΔTyr-EEEEE peptide by $^{18}O$ glutamate.** The LC-MS/MS pipeline was used to analyze tubulin variants that were polyglutamylated by TTLL11 in the presence of isotopically labeled glutamate $^{18}O$. Peaks of interest observed in b- and y-series of the fragmentation spectrum are highlighted in green and magenta, respectively. The fragmentation spectrum of the αΔTyr-EEEEE peptide reveals that the added glutamates are positioned at the very C-terminus of the peptide (and all are α-linked).

**Fig. S12.**

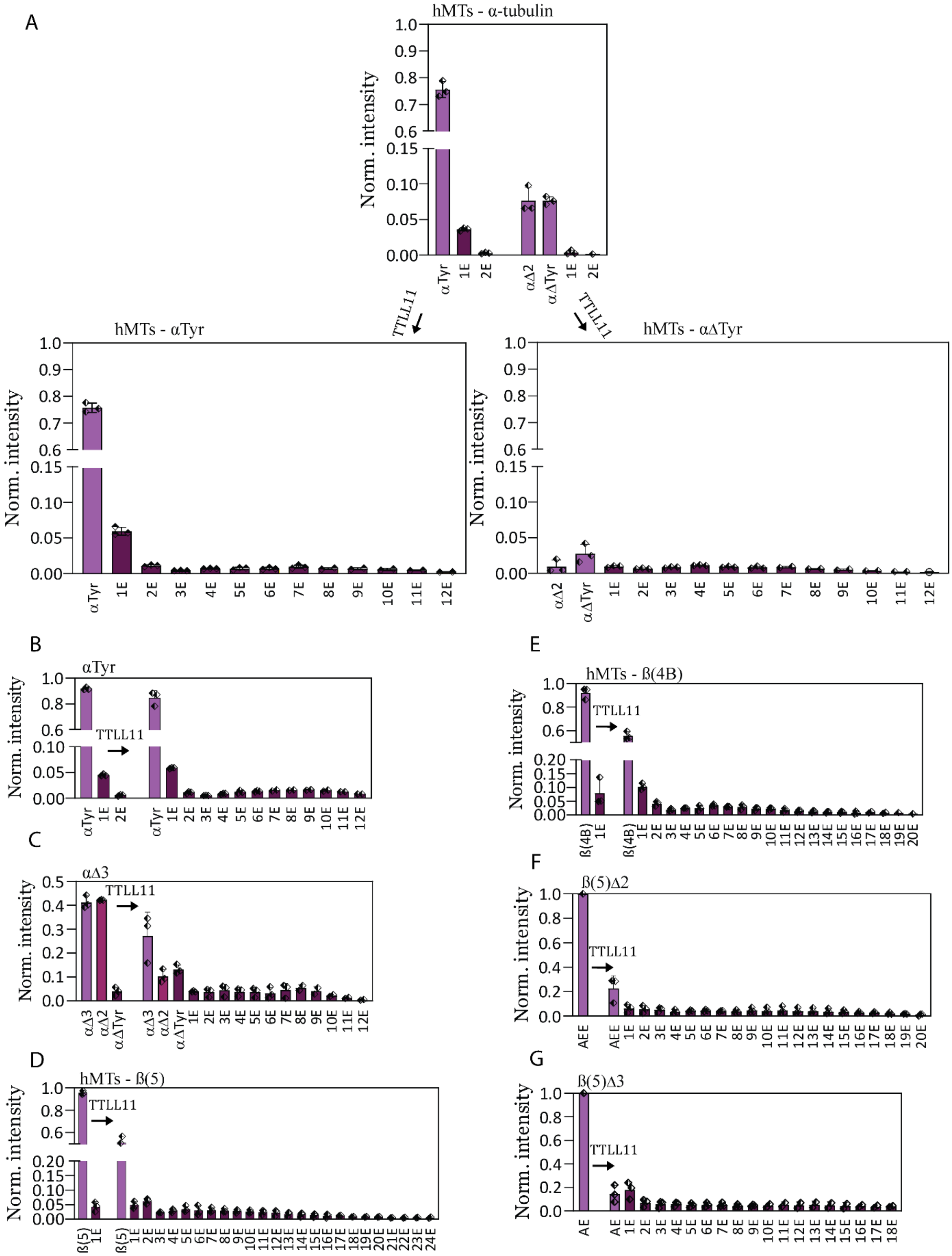


**Fig. S12. The LC-MS quantification of tubulin glutamylation by TTLL11.**
Tubulin variants (in the form of MTs) were incubated with TTLL11 in the presence of isotopically labeled glutamate ($D_5$) and intensities of peptides with and without polyE chains attached were summed. The data are shown as the normalized MS intensities, where the sum of intensities of the peptide pool before and after TTLL11 treatment equals 1 ($n$ = 3, technical replicates). **A. Glutamylation of human α-tubulins isolated from HEK293T cells.** Upper panel –

composition of α-tubulins isolated from HEK293T cells with the majority of the αTyr variant and lesser abundance of αΔTyr/αΔ2 variants. Lower panels – quantification of the individual C-tail peptides with different polyE chains for the αTyr variant (left) and the αΔTyr/αΔ2 variants (right), $n = 3$. While forming a minority of the tubulin substrate αΔTyr/αΔ2 are preferentially modified by TTLL11 compared to the αTyr variant. **B**. **Glutamylation of the αTyr enriched fractions.** The TTL treatment was used to increase the abundance of the αTyr variant to nearly 100% and then polyglutamylated by TTLL11. In the absence of branching, the αTyr variant is almost not modified by TTLL11. **C**. **Glutamylation of the αΔ3/αΔ2 enriched fractions.** The CPA/CCP1 treatment was used to increase the abundance of the αΔ3/αΔ2 variants that were then polyglutamylated by TTLL11. Both αΔ3/αΔ2 are efficiently glutamylated by TTLL11, although glutamylation of αΔ2 is preferred. **D, E. Glutamylation of major β-tubulin variants with intact C-termini.** Either of the β(4B) and β(5) variants is efficiently glutamylated by TTLL11 as approximately 50% of the original peptides are glutamylated with up to 20 glutamate units upon TTLL11 treatment. **F, G. Glutamylation of the β(5)Δ2 and β(5)Δ3 enriched fractions.** The β(5) was modified by the treatment with recombinant ttTMCP resulting in tubulin fractions enriched in β(5)Δ2 and β(5)Δ3 variants that were then polyglutamylated by TTLL11. Glutamylation of these variants is very efficient with the majority of the substrate glutamylated in the reaction mixture (around 80 %).

Fig. S13.

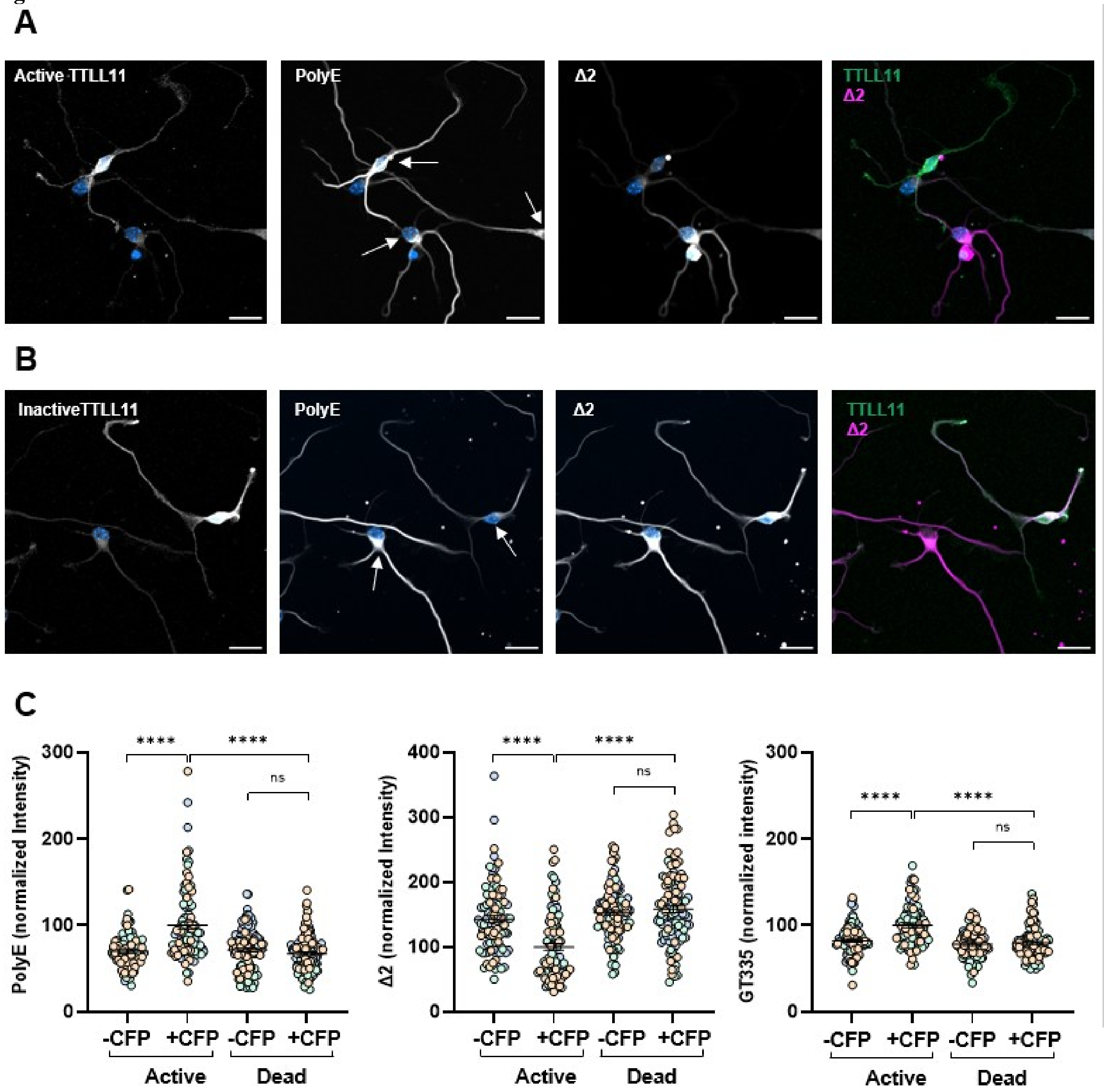


**Fig S13. Effect of ectopic expression of TTLL11 in hippocampal neurons.**
**A,B**. **Confocal images showing representative examples of WT hippocampal neurons** transfected with active (**A**) or inactive (**B**) TTLL11 together with CFP, cultured 2 days *in vitro* and stained for polyE Gre and αΔ2. The CFP signal, amplified using anti-GFP antibody, reveals cells overexpressing the two versions of TTLL11. Arrows indicate neuron cell bodies. Scale bar = 20 µm. **C. Quantification of images of neurons stained for polyE Gre and αΔ2** (as in A,B), or stained with GT335 after transfection with active or inactive TTLL11. Each point represents a different cell, at least 20 positive and negative cells from three independent experiments (orange, blue, green points) were analyzed. Data are represented as mean ± SEM. Statistical significance was determined by two-way ANOVA followed by Sidak's multiple comparison post-test. ns, non-significant, ****$p < 0.0001$. Cells transfected with active TTLL11 show decreased αΔ2 content in comparison to non-transfected cells, together with increased polyE and GT335 signals. These changes are not observed with inactive TTLL11.

**Fig. S14.**

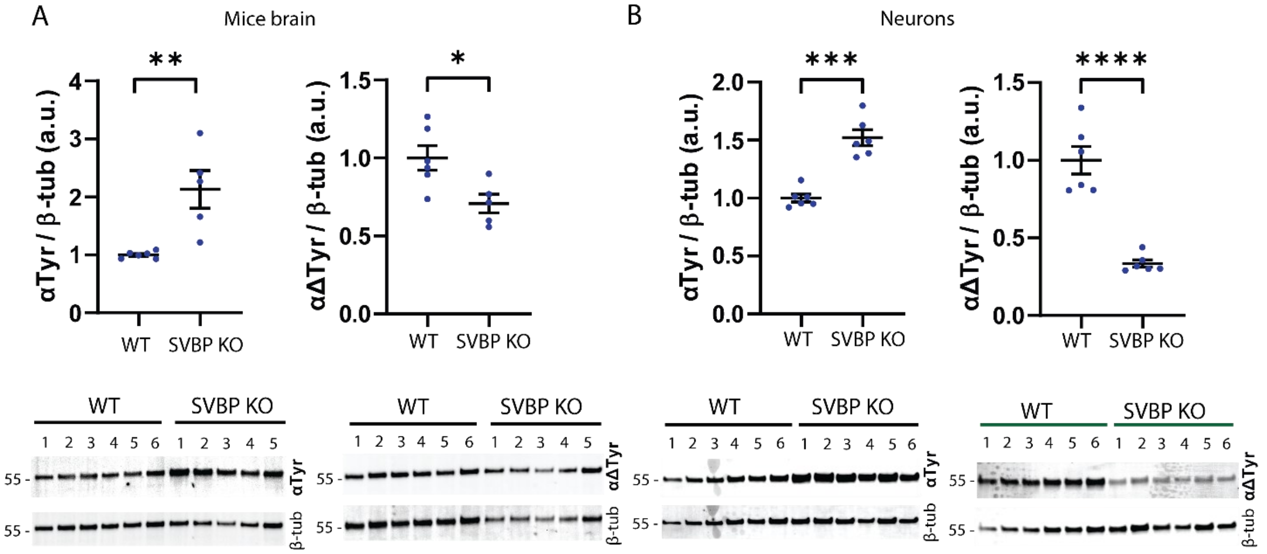


**Fig. S14. αTyr and αΔTyr levels in mice brain and neuron extracts from WT and SVBP KO mice.**
**A,B**. **Immunoblots and their quantification of the ratio of the tubulin modification to β-tubulin** in protein samples from 15-weeks old mice brain (**A**) and cortical neurons cultured 8 days *in vitro* (**B**). The absence of SVBP clearly affects tyrosinated/detyrosinated α-tubulin balance in brain and neurons. In neurons, αTyr is increased 1.5-times and αΔTyr is reduced by 70%, similar differences in the brain. Data represent mean ± SEM. *n* = 6 or 5 animals respectively for WT and SVBP KO, *n* = 6 independent neuronal differentiation experiment for each genotype. Unpaired t-test, ns, non-significant, *$p < 0.05$, **$p<0.01$, ***$p<0.001$, ****$p<0.0001$.

**Fig. S15.**

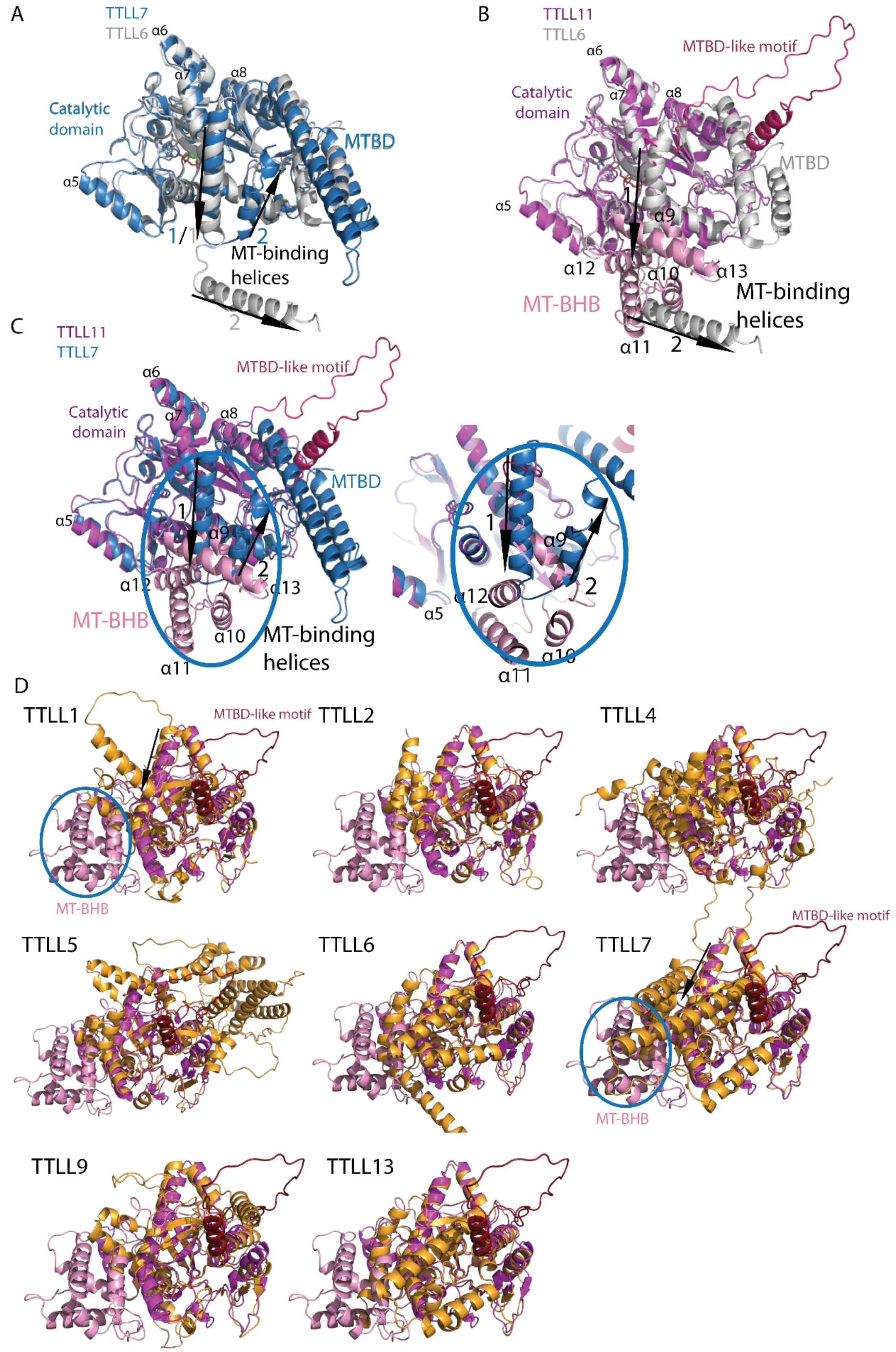


**Fig. S15. Superposition of 3D models of TTLL glutamylases.**

**A-C. Superposition of 3D structures of TTLL11/6/7.** 3D structures of glutamylase pairs TTLL6/7 (A), TTLL11/6 (B), and TTLL11/7 (C) were superimposed on corresponding Cα atoms of their catalytic domain (128 – 466 for TTLL11). Structure model of TTLL6, TTLL7, and TTLL11 (8U3Z (28), provided by Antonina Roll-Mecak (25) and 9HQ4, respectively). The enzymes are shown in cartoon representation and colored grey, blue, and

magenta/hotpink/pink for TTLL6, TTLL7, and TTLL11, respectively. While the structures of catalytic domains are almost identical and superpose well, there are pronounced differences in the structure and positions of putative MT-binding domains/helices. For the TTLL6/7 pair, the helix-loop-helix motifs of the MT-binding domain, which are implicated in MT interactions, are marked by black arrows. The corresponding segment in TTLL11, referred to as the MT-binding domain like motif (MTBD-like), is not visible in the cryo-EM density thus is expected to be flexible and not involved in the MT binding. Instead, the primary TTLL11/MT interface comprises a structurally divergent five-helix bundle (MT-BHB), in which helices α10 and α11 are involved in MT binding. **D. Superposition of 3D models of TTLL glutamylases.** The glutamylases were superimposed on corresponding Cα atoms of their catalytic domains (128 – 466 for TTLL11). The structure of TTLL11 is positioned at 90° left turn from the orientation in A,B, and C. Catalytic domain (magenta), MT-BHB (pink, in blue ellipse labeled for TTLL1 and TTLL7 alignment), MTBD-like motif (dark red). Superposed TTLLs are in orange cartoon representation. While there is substantial structural overlap of the catalytic domains, the MT-BHB is unique for TTLL11 with no structural counterpart in any TTLL polyglutamylase. The predicted internally disordered regiones were deleted for clarity.

**Fig. S16.**

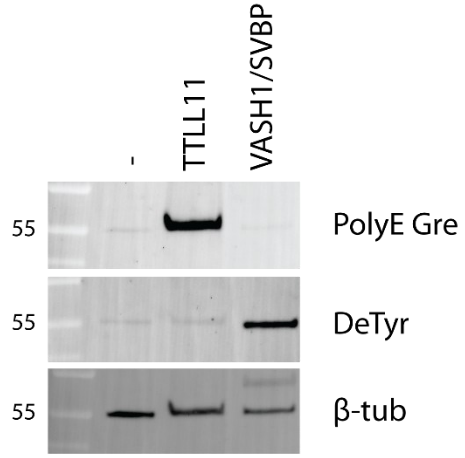


**Fig. S16. Validation of the antibody against polyglutamates developed in guinea pig (polyE Gre).**

HEK293T cells, which have very low level of endogenous polyglutamylation, were used to validate the antibody. Cultures and transfections, as well as SDS-PAGE and immunoblots, were performed as in Aillaud et al *(3)* (JetPRIME transfection reagent), except that fluorescent secondary antibodies were used (anti-rabbit or anti-guinea pig Cyanine-3 (1:2000), and anti-mouse Alexa-488 (1:2000), Jackson Immunoresearch) instead of secondary antibodies conjugated with HRP. Immunoblots of protein extracts from HEK293T cells transfected either with plasmids encoding human VASH1 and SVBP (*3, 74*) or with a plasmid encoding TTLL11 (*18*). The antibody to β-tubulin (β-tub) reveals the amounts of tubulin. Non-transfected cells (-) show endogenous levels of tubulin modifications. The polyE antibody made in Grenoble (polyE Gre) detect a robust band with low antibody concentration (1 :10 000) only in the TTLL11 polyglutamylase overexpressing cells, while it does not detect detyrosinated tubulin generated by the VASH1-SVBP enzyme. In contrast, the anti-detyrosinated tubulin antibody (deTyr-tub, 1 :10 000) (3), reveals the two glutamates of the C-terminus of tubulin generated by VASH1 detyrosination, but does not reveal polyglutamates generated by TTLL11. Thus, the polyE Gre antibody is specific for chain of at least three glutamates, as the polyE used in Van Dijk et al (*18*).

**Fig. S17.1**

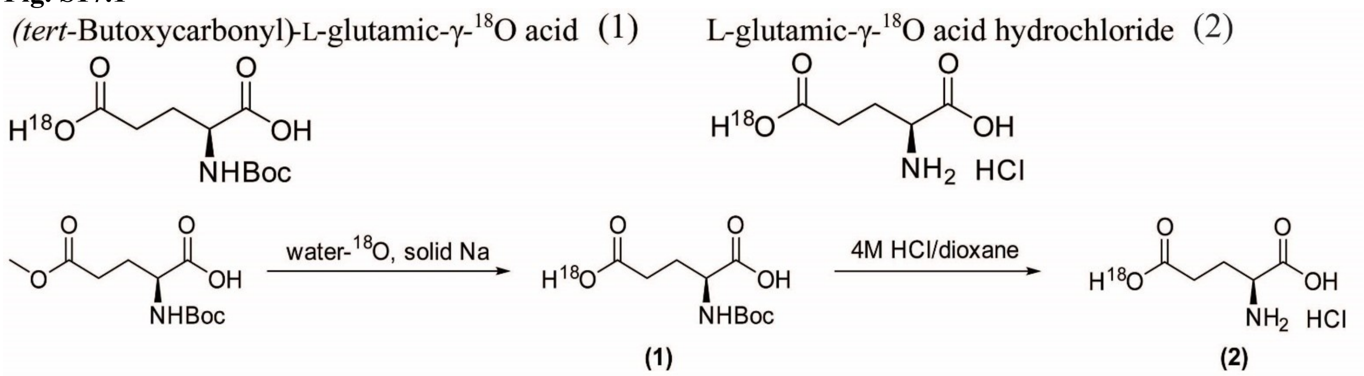


**Fig. S17. Synthesis of $^{18}$O-labeled glutamate.**

A reaction scheme of final steps of L-glutamic-γ-$^{18}$O acid hydrochloride synthesis.

**Table S1 – List of used primers**

| Primers | |
|---|---|
| **Primer name** | **Sequence** |
| hTTLL11_GTW_1F | GAGAACCTGTACTTCCAGGGCGGAGGCACCATGCGGCGGGGCAGCTCCGAG |
| TTLL11 710 GW R | GGGGACCACTTTGTACAAGAAAGCTGGGTTATTA GGACAGGGTATGTCTGGGATGGG |
| hTTLL11_GTW_122F | GAGAACCTGTACTTCCAGGGCGGAGGCGAG AACGGCTCCCAGCGGCCGGTC |
| hTTLL11_GTW_486R | GGGGACCACTTTGTACAAGAAAGCTGGGTT ATTACTTAAGTGGGTCCATGAGGCGCAG |
| mCCP1_1_GW_F | GAGAACCTGTACTTCCAGGGCGGAGGCACC ATGAGCAAGCTAAAAGTGGTG |
| mCCP1_1219_GW_R | GGGGACCACTTTGTACAAGAAAGCTGGGTTATTA AATCAGGTGTGTTCTTGATAC |
| TTL_1_GW_F | GAGAACCTGTACTTCCAGGGCGGAGGCACCATGTACACCTTCGTGGTACGCG |
| TTL_377_GW_R | GGGGACCACTTTGTACAAGAAAGCTGGGTTATTACAGCTTGATGAAGGCAGCTGG |
| hTTLL11_R601E_F | CTCTACATTGACATCACA GAG AGGTGGAACTCC |
| hTTLL11_R601E_R | GGTCATGGAGTTCCACCT CTC TGTGATGTCAATG |
| hTTLL11_I594W_F | GTCCATGGCTGCCG TGG ACTGGCTCTACATTGAC |
| hTTLL11_I594W_R | GTGTGATGTCAATGTAGAG CCA GTCCACGGCAGC |
| Lenti_expr_1_F | GTCTGATCTCAACAAGCTGTCTAGAGAATTC CGACTCACTATAGGGAGACCCAAGC |
| Lenti_expr_R | CAGCTCGACTCAAGAGTCCTCGAGGGCAAACAACAGATGGCTGGCAAC |
| Lenti_expr_ins_F | CTCGAGGACTCTTGAGTCGAGCTG |
| Lenti_expr_ins_R | GAATTCTCTAGACAGCTTGTTGAGATCAGAC |
| Lenti_expr_mid_F | GGTGGATTCCGGTGGTACTGCG |
| Lenti_expr_mid_R | CGCAGTACCACCGGAATCCACC |
| TTLL11_567_stop_F | CTGGGCATCAAGGGGACAATG taa TTGGGGCCAACAGGCTTTCG |
| TTLL11_567_stop_R | CGAAAGCCTGTTGGCCCCAA tta CATTGTCCCCTTGATGCCCAG |

**Table S2 – List of used plasmids**

| Plasmids | | |
|---|---|---|
| **Plasmid name** | **Fusion** | **Reference** |
| pEYFP-spacer-hTTLL11_1-710 | EYFP-hTTLL11 | Kind gift from Carsten Janke |
| pEYFP-spacer-hTTLL11_1-710 E441G | EYFP-hTTLL11 | Kind gift from Carsten Janke |
| TTL+pWPT | mTTL | Marie-Jo Moutin |
| p mVASH2 IRES SVBP | FLAG-VASH2-sfGFP-His-IRES-SVBP | Marie-Jo Moutin |
| pMM322_hTTLL11_1-710 | Twin-Strap-FLAG-HALO-hTTLL11 | This study |
| pMM322_hTTLL11_122-710 | Twin-Strap-FLAG-HALO-hTTLL11 | This study |
| pMM322_hTTLL11_122-486 | Twin-Strap-FLAG-HALO-hTTLL11 | This study |
| pMM322_hTTLL11_212-656 | Twin-Strap-FLAG-HALO-hTTLL11 | This study |
| pMM322_hTTLL11_547-659 | Twin-Strap-FLAG-HALO-hTTLL11 | This study |
| pHR_CMV_TetO2_322_mCCP1 | Twin-Strap-FLAG-HALO-mCCP1 | This study |
| pHR_CMV_TetO2_322_hTTLL11_122-710_E53441G | Twin-Strap-FLAG-HALO-hTTLL11 | This study |
| pMM322_hTTLL11_122-710_E441G | Twin-Strap-FLAG-HALO-hTTLL11 | This study |
| pMM322_hTTLL11_122-710_I594W | Twin-Strap-FLAG-HALO-hTTLL11 | This study |
| pMM322_hTTLL11_122-710_R601E | Twin-Strap-FLAG-HALO-hTTLL11 | This study |

| | | |
|---|---|---|
| pMM322_hTTLL11_122-710_KKKR_EEEE | Twin-Strap-FLAG-HALO-hTTLL11 | This study |
| pEC566_mTTL_1-377 | His-MBP-mTTL | This study |
| pMM322 hTTLL6_1-891 | Twin-Strap-FLAG-HALO-hTTLL6 | This study |
| pMM322 hTTLL7_1-887 | Twin-Strap-FLAG-HALO-hTTLL7 | This study |
| pMM322 ttTMCP_1-491 | Twin-Strap-FLAG-HALO-ttTMCP | This study |

**Table S3 – List of used antibodies**

| **Antibodies** | | | | |
|---|---|---|---|---|
| **Antibody name** | **Source organism** | **Company** | **Dilution** | **RRID** |
| anti-Polyglutamate chain (polyE) | Rabbit | AdiopoGen | 1:4000 | AB_2490540 |
| Anti-alpha Tubulin antibody - Microtubule Marker | Rabbit | Abcam | 1:2000 | AB_2210057 |
| Anti-β-Tubulin antibody | Mouse | Sigma | 1:2000 | AB_477556 |
| Glu-alpha-Tubulin (Detyrosinated alpha-Tubulin) (alpha) Rabbit Polyclonal Antibody | Rabbit | OriGene AP54970SU-N | 1:2000 | |
| Anti-α-Tubulin antibody, tyrosinated, clone YL1/2 | Rat | Sigma | 1:2000 | AB_2890657 |
| anti-polyglutamylation Modification, mAb (GT335) | Mouse | AdipoGen | 1:4000 | AB_2490211 |
| Precision Protein StrepTactin-HRP Conjugate | StrepTactin-HRP | BioRad | 1:4000 | |
| Anti-Rabbit IgG - Peroxidase antibody produced in goat | goat | Sigma | 1:10000 | AB_258284 |
| Anti-Mouse IgG - Peroxidase antibody produced in goat | goat | Sigma | 1:10000 | AB_258167 |
| anti tubulin α3A1 (αtot) | mouse | Moutin Lab | 1:10000 | |
| anti tubulin β3A11 (βtot) | mouse | Moutin Lab | 1:10000 | |
| tubulin α Δ2 | rabbit | Moutin Lab | 1:1000 | |
| anti-GFP | chicken | Sigma | 1:1000 | AB_16901 |
| anti-polyE Gre | guinea pig | Moutin Lab | 1:5000 | |
| mouse conjugated to Alexa-488, | mouse | JACKSON IMMUNORESEARCH | 1:500 | 715-545-150 |
| anti-guinea pig-Cyanine3 | guinea pig | INTERCHIM | 1:500 | 706-165-148 |
| anti-rabbit-Cyanine3 | rabbit | JACKSON IMMUNORESEARCH | 1:500 | 711-165-152 |
| anti-rat-Cyanine | rat | JACKSON IMMUNORESEARCH | 1:500 | 712-165-153 |
| anti-chicken conjugated to Alexa-488 | chicken | JACKSON IMMUNORESEARCH | 1:500 | 703-545-155 |
| anti-mouse conjugated to Alexa-647 | mouse | JACKSON IMMUNORESEARCH | 1:500 | 715-605-150 |
| anti-Polyglutamylation Modification, mAb (GT335) | mouse | AdipoGen | 1:4000 | AB_2490210 |

**Table S4 - Cryo-EM data collection, model refinement and validation statistics.**

| | |
|---|---|
| **Data collection** | |
| Microscope | Titan Krios (G1-2) |
| Detector | Falcon 3EC direct electron camera |
| Voltage (kV) | 300 |
| Defocus range | -0.8 - -2 µm |
| Pixel size | 1.349 Å |
| Frames per movie | 30 |
| Movies collected | 8.8k |
| **Image processing** | |
| Symmetry | C1 |
| Initial particle images | 250k MT segments |
| Final particles images | 240k subtracted segments |
| Map resolution | 3.2 Å |
| FSC threshold | 0.143 |
| Map resolution range | 3 to 11 Å |
| **Model refinement and validation statistics** | |
| Atomic modeling refinements packages | Coot, Phenix |
| Initial model used | AF-Q8NHH1-F1-v4 |
| Model Resolution (Å) | 3.2 |
| FSC threshold | 0.143 |
| **Model composition** | |
| Non-hydrogen atoms | 17592 |
| Protein residues | 2230 |
| **Ligands** | 4 GMPCPP, 4 $Mg^{2+}$ |
| **B factor ($Å^2$)** | 102.3 |

## REFERENCES AND NOTES


1. A. Roll-Mecak, How cells exploit tubulin diversity to build functional cellular microtubule mosaics. *Curr. Opin. Cell Biol.* **56**, 102–108 (2019).

2. A. Roll-Mecak, The tubulin code in microtubule dynamics and information encoding. *Dev. Cell* **54**, 7–20 (2020).

3. C. Aillaud, C. Bosc, L. Peris, A. Bosson, P. Heemeryck, J. Van Dijk, J. Le Friec, B. Boulan, F. Vossier, L. E. Sanman, S. Syed, N. Amara, Y. Coute, L. Lafanechere, E. Denarier, C. Delphin, L. Pelletier, S. Humbert, M. Bogyo, A. Andrieux, K. Rogowski, M.-J. Moutin, Vasohibins/SVBP are tubulin carboxypeptidases (TCPs) that regulate neuron differentiation. *Science* **358**, 1448–1453 (2017).

4. J. Nieuwenhuis, A. Adamopoulos, O. B. Bleijerveld, A. Mazouzi, E. Stickel, P. Celie, M. Altelaar, P. Knipscheer, A. Perrakis, V. A. Blomen, T. R. Brummelkamp, Vasohibins encode tubulin detyrosinating activity. *Science* **358**, 1453–1456 (2017).

5. L. Landskron, J. Bak, A. Adamopoulos, K. Kaplani, M. Moraiti, L. G. van den Hengel, J.-Y. Song, O. B. Bleijerveld, J. Nieuwenhuis, T. Heidebrecht, L. Henneman, M.-J. Moutin, M. Barisic, S. Taraviras, A. Perrakis, T. R. Brummelkamp, Posttranslational modification of microtubules by the MATCAP detyrosinase. *Science* **376**, eabn6020 (2022).

6. S. Nicot, G. Gillard, H. Impheng, E. Joachimiak, S. Urbach, K. Mochizuki, D. Wloga, F. Juge, K. Rogowski, A family of carboxypeptidases catalyzing α- and β-tubulin tail processing and deglutamylation. *Sci. Adv.* **9**, eadi7838 (2023).

7. K. Rogowski, J. van Dijk, M. M. Magiera, C. Bosc, J.-C. Deloulme, A. Bosson, L. Peris, N. D. Gold, B. Lacroix, M. B. Grau, N. Bec, C. Larroque, S. Desagher, M. Holzer, A. Andrieux, M.-J. Moutin, C. Janke, A family of protein-deglutamylating enzymes associated with neurodegeneration. *Cell* **143**, 564–578 (2010).

8. O. Tort, S. Tanco, C. Rocha, I. Bieche, C. Seixas, C. Bosc, A. Andrieux, M. J. Moutin, F. X. Aviles, J. Lorenzo, C. Janke, The cytosolic carboxypeptidases CCP2 and CCP3 catalyze posttranslational removal of acidic amino acids. *Mol. Biol. Cell* **25**, 3017–3027 (2014).

9. D. Raybin, M. Flavin, An enzyme tyrosylating α-tubulin and its role in microtubule assembly. *Biochem. Biophys. Res. Commun.* **65**, 1088–1095 (1975).

10. L. Paturle-Lafanechere, M. Manier, N. Trigault, F. Pirollet, H. Mazarguil, D. Job, Accumulation of delta 2-tubulin, a major tubulin variant that cannot be tyrosinated, in neuronal tissues and in stable microtubule assemblies. *J. Cell Sci.* **107**, 1529–1543 (1994).

11. C. Aillaud, C. Bosc, Y. Saoudi, E. Denarier, L. Peris, L. Sago, N. Taulet, A. Cieren, O. Tort, M. M. Magiera, C. Janke, V. Redeker, A. Andrieux, M.-J. Moutin, K. S. Bloom, Evidence for new C-terminally truncated variants of α- and β-tubulins. *Mol. Biol. Cell* **27**, 640–653 (2016).

12. M. M. Magiera, P. Singh, S. Gadadhar, C. Janke, Tubulin posttranslational modifications and emerging links to human disease. *Cell* **173**, 1323–1327 (2018).

13. J. E. Lee, J. L. Silhavy, M. S. Zaki, J. Schroth, S. L. Bielas, S. E. Marsh, J. Olvera, F. Brancati, M. Iannicelli, K. Ikegami, A. M. Schlossman, B. Merriman, T. Attie-Bitach, C. V. Logan, I. A. Glass, A. Cluckey, C. M. Louie, J. H. Lee, H. R. Raynes, I. Rapin, I. P. Castroviejo, M. Setou, C. Barbot, E. Boltshauser, S. F. Nelson, F. Hildebrandt, C. A. Johnson, D. A. Doherty, E. M. Valente, J. G. Gleeson, CEP41 is mutated in Joubert syndrome and is required for tubulin glutamylation at the cilium. *Nat. Genet.* **44**, 193–199 (2012).

14. P. Xia, B. Ye, S. Wang, X. Zhu, Y. Du, Z. Xiong, Y. Tian, Z. Fan, Glutamylation of the DNA sensor cGAS regulates its binding and synthase activity in antiviral immunity. *Nat. Immunol.* **17**, 369–378 (2016).

15. A. Deshpande, J. Brants, C. Wasylyk, O. van Hooij, G. W. Verhaegh, P. Maas, J. A. Schalken, B. Wasylyk, TTLL12 has a potential oncogenic activity, suppression of ligation of nitrotyrosine to the C-terminus of detyrosinated α-tubulin, that can be overcome by molecules identified by screening a compound library. *PLOS ONE* **19**, e0296960 (2024).

16. L. Froidevaux-Klipfel, B. Targa, I. Cantaloube, H. Ahmed-Zaid, C. Pous, A. Baillet, Septin cooperation with tubulin polyglutamylation contributes to cancer cell adaptation to taxanes. *Oncotarget* **6**, 36063–36080 (2015).

17. I. Zadra, S. Jimenez-Delgado, M. Anglada-Girotto, C. Segura-Morales, Z. J. Compton, C. Janke, L. Serrano, V. Ruprecht, I. Vernos, Chromosome segregation fidelity requires microtubule polyglutamylation by the cancer downregulated enzyme TTLL11. *Nat. Commun.* **13**, 7147 (2022).

18. J. van Dijk, K. Rogowski, J. Miro, B. Lacroix, B. Eddé, C. Janke, A targeted multienzyme mechanism for selective microtubule polyglutamylation. *Mol. Cell* **26**, 437–448 (2007).

19. M. Kravec, O. Šedo, J. Nedvědová, M. Micka, M. Šulcová, N. Zezula, K. Gömöryová, D. Potěšil, R. S. Ganji, S. Bologna, I. Červenka, Z. Zdráhal, J. Harnoš, K. Tripsianes, C. Janke, C. Bařinka, V. Bryja, Carboxy-terminal polyglutamylation regulates signaling and phase separation of the Dishevelled protein. *EMBO J.* **43**, 5635–5666 (2024).

20. B. M. Lorton, C. Warren, H. Ilyas, P. Nandigrami, S. Hegde, S. Cahill, S. M. Lehman, J. Shabanowitz, D. F. Hunt, A. Fiser, D. Cowburn, D. Shechter, Glutamylation of Npm2 and Nap1 acidic disordered regions increases DNA mimicry and histone chaperone efficiency. *iScience* **27**, 109458 (2024).

21. J. van Dijk, J. Miro, J. M. Strub, B. Lacroix, A. van Dorsselaer, B. Edde, C. Janke, Polyglutamylation is a post-translational modification with a broad range of substrates. *J. Biol. Chem.* **283**, 3915–3922 (2008).

22. B. Lacroix, J. van Dijk, N. D. Gold, J. Guizetti, G. Aldrian-Herrada, K. Rogowski, D. W. Gerlich, C. Janke, Tubulin polyglutamylation stimulates spastin-mediated microtubule severing. *J. Cell Biol.* **189**, 945–954 (2010).

23. R. O'Hagan, M. Silva, K. C. Q. Nguyen, W. Zhang, S. Bellotti, Y. H. Ramadan, D. H. Hall, M. M. Barr, Glutamylation regulates transport, specializes function, and sculpts the structure of cilia. *Curr. Biol.* **27**, 3430–3441.e6 (2017).

24. H. Mathieu, S. A. Patten, J. A. Aragon-Martin, L. Ocaka, M. Simpson, A. Child, F. Moldovan, Genetic variant of TTLL11 gene and subsequent ciliary defects are associated with idiopathic scoliosis in a 5-generation UK family. *Sci. Rep.* **11**, 11026 (2021).

25. C. P. Garnham, A. Vemu, E. M. Wilson-Kubalek, I. Yu, A. Szyk, G. C. Lander, R. A. Milligan, A. Roll-Mecak, Multivalent microtubule recognition by tubulin tyrosine ligase-like family glutamylases. *Cell* **161**, 1112–1123 (2015).

26. K. K. Mahalingan, E. Keith Keenan, M. Strickland, Y. Li, Y. Liu, H. L. Ball, M. E. Tanner, N. Tjandra, A. Roll-Mecak, Structural basis for polyglutamate chain initiation and elongation by TTLL family enzymes. *Nat. Struct. Mol. Biol.* **27**, 802–813 (2020).

27. G. Fu, S. Yan, C. J. Khoo, V. C. Chao, Z. Liu, M. Mukhi, R. Hervas, X. D. Li, S.-C. Ti, Integrated regulation of tubulin tyrosination and microtubule stability by human α-tubulin isotypes. *Cell Rep.* **42**, 112653 (2023).

28. K. K. Mahalingan, D. A. Grotjahn, Y. Li, G. C. Lander, E. A. Zehr, A. Roll-Mecak, Structural basis for α-tubulin-specific and modification state-dependent glutamylation. *Nat. Chem. Biol.* **20**, 1493–1504 (2024).

29. L. A. Amos, Microtubule structure and its stabilisation. *Org. Biomol. Chem.* **2**, 2153–2160 (2004).

30. ProteinAtlas, "Created with The Human Protein Atlas" (2024); www.proteinatlas.org/.

31. C. Sanyal, N. Pietsch, S. Ramirez Rios, L. Peris, L. Carrier, M. J. Moutin, The detyrosination/re-tyrosination cycle of tubulin and its role and dysfunction in neurons and cardiomyocytes. *Semin. Cell Dev. Biol.* **137**, 46–62 (2023).

32. C. Janke, M. M. Magiera, The tubulin code and its role in controlling microtubule properties and functions. *Nat. Rev. Mol. Cell Biol.* **21**, 307–326 (2020).

33. K. Natarajan, S. Gadadhar, J. Souphron, M. M. Magiera, C. Janke, Molecular interactions between tubulin tails and glutamylases reveal determinants of glutamylation patterns. *EMBO Rep.* **18**, 1013–1026 (2017).

34. S. Bodakuntla, A. Schnitzler, C. Villablanca, C. Gonzalez-Billault, I. Bieche, C. Janke, M. M. Magiera, Tubulin polyglutamylation is a general traffic-control mechanism in hippocampal neurons. *J. Cell Sci.* **133**, jcs241802 (2020).

35. A. T. Pagnamenta, P. Heemeryck, H. C. Martin, C. Bosc, L. Peris, I. Uszynski, S. Gory-Fauré, S. Couly, C. Deshpande, A. Siddiqui, A. A. Elmonairy, WGS500 Consortium, Genomics England Research Consortium, S. Jayawant, S. Murthy, I. Walker, L. Loong, P. Bauer, F. Vossier, E. Denarier, T. Maurice, E. L. Barbier, J.-C. Deloulme, J. C. Taylor, E. M. Blair, A. Andrieux, M.-J. Moutin, Defective tubulin detyrosination causes structural brain abnormalities with cognitive deficiency in humans and mice. *Hum. Mol. Genet.* **28**, 3391–3405 (2019).

36. M. M. Magiera, S. Bodakuntla, J. Ziak, S. Lacomme, P. Marques Sousa, S. Leboucher, T. J. Hausrat, C. Bosc, A. Andrieux, M. Kneussel, M. Landry, A. Calas, M. Balastik, C. Janke, Excessive tubulin polyglutamylation causes neurodegeneration and perturbs neuronal transport. *EMBO J.* **37**, e100440 (2018).

37. B. Badarudeen, H.-J. Chiang, L. Collado, L. Wang, I. Sanchez, B. D. Dynlacht, The tubulin poly-glutamylase complex, TPGC, is required for phosphatidyl inositol homeostasis and cilium assembly and maintenance. bioRxiv 2025.03.03.641315 [Preprint] (2025). https://doi.org/10.1101/2025.03.03.641315.

38. C. Janke, K. Rogowski, D. Wloga, C. Regnard, A. V. Kajava, J.-M. Strub, N. Temurak, J. van Dijk, D. Boucher, A. van Dorsselaer, S. Suryavanshi, J. Gaertig, B. Eddé, Tubulin polyglutamylase enzymes are members of the TTL domain protein family. *Science* **308**, 1758–1762 (2005).

39. A. Konietzny, Y. Han, Y. Popp, B. van Bommel, A. Sharma, P. Delagrange, N. Arbez, M. J. Moutin, L. Peris, M. Mikhaylova, Efficient axonal transport of endolysosomes relies on the balanced ratio of microtubule tyrosination and detyrosination. *J. Cell Sci.* **137**, jcs261737 (2024).

40. J. J. Nirschl, M. M. Magiera, J. E. Lazarus, C. Janke, E. L. Holzbaur, α-Tubulin tyrosination and CLIP-170 phosphorylation regulate the initiation of dynein-driven transport in neurons. *Cell Rep.* **14**, 2637–2652 (2016).

41. L. Peris, M. Thery, J. Faure, Y. Saoudi, L. Lafanechere, J. K. Chilton, P. Gordon-Weeks, N. Galjart, M. Bornens, L. Wordeman, J. Wehland, A. Andrieux, D. Job, Tubulin tyrosination is

a major factor affecting the recruitment of CAP-Gly proteins at microtubule plus ends. *J. Cell Biol.* **174**, 839–849 (2006).

42. L. Peris, M. Wagenbach, L. Lafanechere, J. Brocard, A. T. Moore, F. Kozielski, D. Job, L. Wordeman, A. Andrieux, Motor-dependent microtubule disassembly driven by tubulin tyrosination. *J. Cell Biol.* **185**, 1159–1166 (2009).

43. M. O. Steinmetz, A. Akhmanova, Capturing protein tails by CAP-Gly domains. *Trends Biochem. Sci.* **33**, 535–545 (2008).

44. Y.-M. Lu, S. Yan, S.-C. Ti, C. Zheng, Editing of endogenous tubulins reveals varying effects of tubulin posttranslational modifications on axonal growth and regeneration. *eLife* **13**, RP94583 (2024).

45. N. Pathak, T. Obara, S. Mangos, Y. Liu, I. A. Drummond, The zebrafish fleer gene encodes an essential regulator of cilia tubulin polyglutamylation. *Mol. Biol. Cell* **18**, 4353–4364 (2007).

46. A. M. Sheikh, S. Tabassum, Potential role of tubulin glutamylation in neurodegenerative diseases. *Neural Regen. Res.* **19**, 1191–1192 (2024).

47. D. T. Martin, N. Jardin, J. Vougny, F. Giudicelli, L. Gasmi, N. Berbée, V. Henriot, L. Lebrun, C. Haumaître, M. Kneussel, X. Nicol, C. Janke, M. M. Magiera, J. Hazan, C. Fassier, Tubulin glutamylation regulates axon guidance via the selective tuning of microtubule-severing enzymes. *EMBO J.* **44**, 107–140 (2025).

48. S. Chakraborti, K. Natarajan, J. Curiel, C. Janke, J. Liu, The emerging role of the tubulin code: From the tubulin molecule to neuronal function and disease. *Cytoskeleton* **73**, 521–550 (2016).

49. S. Gadadhar, S. Bodakuntla, K. Natarajan, C. Janke, The tubulin code at a glance. *J. Cell Sci.* **130**, 1347–1353 (2017).

50. E. D. McKenna, S. L. Sarbanes, S. W. Cummings, A. Roll-Mecak, The tubulin code, from molecules to health and disease. *Annu. Rev. Cell Dev. Biol.* **39**, 331–361 (2023).

51. G. A. Viar, G. Pigino, Tubulin posttranslational modifications through the lens of new technologies. *Curr. Opin. Cell Biol.* **88**, 102362 (2024).

52. M. Sirajuddin, L. M. Rice, R. D. Vale, Regulation of microtubule motors by tubulin isotypes and post-translational modifications. *Nat. Cell Biol.* **16**, 335–344 (2014).

53. M. L. Valenstein, A. Roll-Mecak, Graded control of microtubule severing by tubulin glutamylation. *Cell* **164**, 911–921 (2016).

54. L. Skultetyova, K. Ustinova, Z. Kutil, Z. Novakova, J. Pavlicek, J. Mikesova, D. Trapl, P. Baranova, B. Havlinova, M. Hubalek, Z. Lansky, C. Barinka, Human histone deacetylase 6 shows strong preference for tubulin dimers over assembled microtubules. *Sci. Rep.* **7**, 11547 (2017).

55. J. Elegheert, E. Behiels, B. Bishop, S. Scott, R. E. Woolley, S. C. Griffiths, E. F. X. Byrne, V. T. Chang, D. I. Stuart, E. Y. Jones, C. Siebold, A. R. Aricescu, Lentiviral transduction of mammalian cells for fast, scalable and high-level production of soluble and membrane proteins. *Nat. Protoc.* **13**, 2991–3017 (2018).

56. Z. Kutil, L. Skultetyova, D. Rauh, M. Meleshin, I. Snajdr, Z. Novakova, J. Mikesova, J. Pavlicek, M. Hadzima, P. Baranova, B. Havlinova, P. Majer, M. Schutkowski, C. Barinka, The unraveling of substrate specificity of histone deacetylase 6 domains using acetylome peptide microarrays and peptide libraries. *FASEB J.* **33**, 4035–4045 (2019).

57. J. Souphron, S. Bodakuntla, A. S. Jijumon, G. Lakisic, A. M. Gautreau, C. Janke, M. M. Magiera, Purification of tubulin with controlled post-translational modifications by polymerization-depolymerization cycles. *Nat. Protoc.* **14**, 1634–1660 (2019).

58. M. Castoldi, A. V. Popov, Purification of brain tubulin through two cycles of polymerization-depolymerization in a high-molarity buffer. *Protein Expr. Purif.* **32**, 83–88 (2003).

59. K. Ustinova, Z. Novakova, M. Saito, M. Meleshin, J. Mikesova, Z. Kutil, P. Baranova, B. Havlinova, M. Schutkowski, P. Matthias, C. Barinka, The disordered N-terminus of HDAC6

is a microtubule-binding domain critical for efficient tubulin deacetylation. *J. Biol. Chem.* **295**, 2614–2628 (2020).

60. M. M. Magiera, C. Janke, Investigating tubulin posttranslational modifications with specific antibodies. *Methods Cell Biol.* **115**, 247–267 (2013).

61. M. Mahamdeh, S. Simmert, A. Luchniak, E. Schaffer, J. Howard, Label-free high-speed wide-field imaging of single microtubules using interference reflection microscopy. *J. Microsc.* **272**, 60–66 (2018).

62. J. Schindelin, I. Arganda-Carreras, E. Frise, V. Kaynig, M. Longair, T. Pietzsch, S. Preibisch, C. Rueden, S. Saalfeld, B. Schmid, J. Y. Tinevez, D. J. White, V. Hartenstein, K. Eliceiri, P. Tomancak, A. Cardona, Fiji: An open-source platform for biological-image analysis. *Nat. Methods* **9**, 676–682 (2012).

63. M. Braun, Z. Lansky, G. Fink, F. Ruhnow, S. Diez, M. E. Janson, Adaptive braking by Ase1 prevents overlapping microtubules from sliding completely apart. *Nat. Cell Biol.* **13**, 1259–1264 (2011).

64. A. A. Hyman, Preparation of marked microtubules for the assay of the polarity of microtubule-based motors by fluorescence. *J. Cell Sci. Suppl.* **14**, 125–127 (1991).

65. A. D. Cook, S. W. Manka, S. Wang, C. A. Moores, J. Atherton, A microtubule RELION-based pipeline for cryo-EM image processing. *J. Struct. Biol.* **209**, 107402 (2020).

66. S. H. W. Scheres, RELION: Implementation of a Bayesian approach to cryo-EM structure determination. *J. Struct. Biol.* **180**, 519–530 (2012).

67. A. Punjani, J. L. Rubinstein, D. J. Fleet, M. A. Brubaker, cryoSPARC: Algorithms for rapid unsupervised cryo-EM structure determination. *Nat. Methods* **14**, 290–296 (2017).

68. R. Sanchez-Garcia, J. Gomez-Blanco, A. Cuervo, J. M. Carazo, C. O. S. Sorzano, J. Vargas, DeepEMhancer: A deep learning solution for cryo-EM volume post-processing. *Commun. Biol.* **4**, 874 (2021).

69. P. Emsley, B. Lohkamp, W. G. Scott, K. Cowtan, Features and development of Coot. *Acta Crystallogr. D Biol. Crystallogr.* **66**, 486–501 (2010).

70. E. F. Pettersen, T. D. Goddard, C. C. Huang, E. C. Meng, G. S. Couch, T. I. Croll, J. H. Morris, T. E. Ferrin, UCSF ChimeraX: Structure visualization for researchers, educators, and developers. *Protein Sci.* **30**, 70–82 (2021).

71. P. D. Adams, P. V. Afonine, G. Bunkoczi, V. B. Chen, I. W. Davis, N. Echols, J. J. Headd, L. W. Hung, G. J. Kapral, R. W. Grosse-Kunstleve, A. J. McCoy, N. W. Moriarty, R. Oeffner, R. J. Read, D. C. Richardson, J. S. Richardson, T. C. Terwilliger, P. H. Zwart, PHENIX: A comprehensive Python-based system for macromolecular structure solution. *Acta Crystallogr. D Biol. Crystallogr.* **66**, 213–221 (2010).

72. V. B. Chen, W. Bryan Arendall III, J. J. Headd, D. A. Keedy, R. M. Immormino, G. J. Kapral, L. W. Murray, J. S. Richardson, D. C. Richardson, MolProbity: All-atom structure validation for macromolecular crystallography. *Acta Crystallogr. D Biol. Crystallogr.* **66**, 12–21 (2010).

73. P. Bankhead, M. B. Loughrey, J. A. Fernandez, Y. Dombrowski, D. G. McArt, P. D. Dunne, S. McQuaid, R. T. Gray, L. J. Murray, H. G. Coleman, J. A. James, M. Salto-Tellez, P. W. Hamilton, QuPath: Open source software for digital pathology image analysis. *Sci. Rep.* **7**, 16878 (2017).

74. N. Wang, C. Bosc, S. Ryul Choi, B. Boulan, L. Peris, N. Olieric, H. Bao, F. Krichen, L. Chen, A. Andrieux, V. Olieric, M. J. Moutin, M. O. Steinmetz, H. Huang, Structural basis of tubulin detyrosination by the vasohibin-SVBP enzyme complex. *Nat. Struct. Mol. Biol.* **26**, 571–582 (2019).
